# Discovery of novel potent ΔF508-CFTR correctors that target the nucleotide binding domain

**DOI:** 10.1002/emmm.201302699

**Published:** 2013-08-27

**Authors:** Norbert Odolczyk, Janine Fritsch, Caroline Norez, Nathalie Servel, Melanie Faria da Cunha, Sara Bitam, Anna Kupniewska, Ludovic Wiszniewski, Julien Colas, Krzysztof Tarnowski, Danielle Tondelier, Ariel Roldan, Emilie L Saussereau, Patricia Melin-Heschel, Grzegorz Wieczorek, Gergely L Lukacs, Michal Dadlez, Grazyna Faure, Harald Herrmann, Mario Ollero, Frédéric Becq, Piotr Zielenkiewicz, Aleksander Edelman

**Affiliations:** 1 Department of Bioinformatics, Institute of Biochemistry and Biophysics, Polish Academy of SciencesWarszawa, Poland; 2 INSERM, U845Paris, France; 3 Faculté de Médecine, Université Paris DescartesParis, France; 4 Université de Poitiers, Institut de Physiologie et Biologie CellulairesPoitiers, France; 5 Epithelix SARL, CH-1228 Plan-Les-OuatesGeneva, Switzerland; 6 Laboratory of Mass Spectrometry, Institute of Biochemistry and Biophysics, Polish Academy of SciencesWarszawa, Poland; 7 Department of Physiology, McGill UniversityMontreal, Canada; 8 Unité Récepteurs-Canaux; Institut Pasteur, CNRSParis, France; 9 Department of Molecular Genetics, German Cancer Research CenterHeidelberg, Germany; 10 INSERM, U955, Equipe 21Creteil, France; 11 Laboratory of Plant Molecular Biology, Faculty of Biology, Warsaw UniversityWarszawa, Poland

**Keywords:** CFTR, chloride channel, cystic fibrosis, drug discovery, ΔF508-CFTR correctors

## Abstract

The deletion of Phe508 (ΔF508) in the first nucleotide binding domain (NBD1) of CFTR is the most common mutation associated with cystic fibrosis. The ΔF508-CFTR mutant is recognized as improperly folded and targeted for proteasomal degradation. Based on molecular dynamics simulation results, we hypothesized that interaction between ΔF508-NBD1 and housekeeping proteins prevents ΔF508-CFTR delivery to the plasma membrane. Based on this assumption we applied structure-based virtual screening to identify new low-molecular-weight compounds that should bind to ΔF508-NBD1 and act as protein–protein interaction inhibitors. Using different functional assays for CFTR activity, we demonstrated that *in silico*-selected compounds induced functional expression of ΔF508-CFTR in transfected HeLa cells, human bronchial CF cells in primary culture, and in the nasal epithelium of homozygous ΔF508-CFTR mice. The proposed compounds disrupt keratin8-ΔF508-CFTR interaction in ΔF508-CFTR HeLa cells. Structural analysis of ΔF508-NBD1 in the presence of these compounds suggests their binding to NBD1. We conclude that our strategy leads to the discovery of new compounds that are among the most potent correctors of ΔF508-CFTR trafficking defect known to date.

→ See accompanying article http://dx.doi.org/10.1002/emmm.201303301

## INTRODUCTION

Cystic fibrosis (CF) is a fatal autosomal recessive genetic disorder caused by loss-of-function mutations in the *CFTR* gene, which encodes the CFTR protein (CF transmembrane conductance regulator) (Riordan et al, 1989). The most frequent mutation in the *CFTR* gene classified to date (http://www.genet.sickkids.on.ca/) is the deletion of phenylalanine 508 in NBD1 domain (ΔF508-CFTR), which is responsible for a severe form of CF (Riordan et al, 1989). CFTR, a PKA-activated Cl^−^ channel, is a rate-limiting factor for fluid absorption in numerous epithelia, such as the lung, pancreas, intestine and sweat glands (Robert et al, 2008). CFTR regulates different ion transports, including Cl^−^/HCO_3_^−^ secretion, by interacting with SLC26An transporters (Ko et al, 2004; Rode et al, 2012), and Na^+^ absorption, possibly by interacting with the epithelial sodium channel ENaC (Berdiev et al, 2009).

The *CFTR*-coded 1480-amino acid protein, which shares structural and folding features with members of the ATP-binding cassette (ABC) transporters, consists of two nucleotide binding domains (NBDs), two transmembrane domains (TMDs) and one mostly unstructured regulatory domain (RD). The latter is specific for CFTR and its functions (Ollero et al, 2006).

ΔF508-CFTR has a reduced ability to escape from the endoplasmic reticulum (ER) and undergoes premature degradation in a proteasome-dependent manner (Jensen et al, 1995; Ward et al, 1995; Younger et al, 2006). Thus, a significant decrease in functional CFTR expression occurs at the apical plasma membrane, which has been defined as a result of a trafficking or folding defect (Carlile et al, 2007). Moreover, a residual amount of ΔF508-CFTR, which in some conditions reaches its native destination, exhibits a dysfunction associated with lower activity, termed a gating defect (Dalemans et al, 1991).

Since the discovery of mutations in *CFTR* as the cause of CF, a number of studies have been conducted to find a pharmacological approach to correct the dysfunction of the mutated proteins (Becq et al, 2011). For missense mutations, such as ΔF508-CFTR, small molecules (correctors) need to facilitate trafficking and delivery of the abnormal protein to the plasma membrane and/or to improve its channel gating (potentiators) (Riordan, 2008). A successful example of potentiator is a VX-770/Ivacaftor, which ameliorates significantly the clinical status of CF patients bearing the G551D mutation and shows no major side effects (Ramsey et al, 2011; Yu et al, 2012).

On the other hand, the fact that protein folding and trafficking are complex, multistep processes involving multiple cellular targets significantly complicates the task of development of ΔF508-CFTR correctors (Kalid et al, 2010; Pedemonte et al, 2005b). Indeed, such molecules are decisively in the minority among the known CFTR modulators.

The hypothesis driven approach has led to the discovery of small molecules, such as phosphodiesterase-type 5 (PDE5) inhibitors (Dormer et al, 2005) (e.g. sildenafil), alpha-glucosidase inhibitors (Norez et al, 2006) (e.g. miglustat) and histone deacetylase-7 inhibitors (Hutt et al, 2010) (e.g. SAHA), that interact with proteins responsible for ΔF508-CFTR processing and increase the amount of ΔF508-CFTR at the plasma membrane. Other molecules such as curcumin or resveratrol derived from plants may act by modifying keratin 18 (K18) network (Hamdaoui et al, 2011; Lipecka et al, 2006). K18 heterodimerizes with keratin 8 (K8) and further evidence suggests that both proteins play a role in the trafficking of CFTR/ΔF508-CFTR (Colas et al, 2012; Duan et al, 2012). Accordingly, we have reported that a decrease in K8 expression leads to functional correction of ΔF508-CFTR (Colas et al, 2012).

Discovery of the high-throughput screening approach has resulted in a significant increase in the number of compounds found to be able to correct the ΔF508-CFTR trafficking defect. Among them are many distinct chemical classes like aminoarylthiazoles, quinazolinylaminopyrimidinones, bisaminomethylbithiazoles (e.g. Corr-4a) (Pedemonte et al, 2005b), 1,4-dihydropiridines (Pedemonte et al, 2005a), quinazolines (e.g. VRT-325) (Loo et al, 2005), the sildenafil analogues like KM11060 (Robert et al, 2008) or galfenine (Robert et al, 2010), and up to date the most potent corrector VX-809 (Van Goor et al, 2011). The mechanism of action for these compounds is not precisely known, which dramatically decreases the chance for further rational development.

A commonly accepted hypothesis postulates that some correctors (e.g. VRT-325, MPB, Corr-4a and the dual activity molecule VRT-532) interact directly with ΔF508-CFTR by stabilising its structure, to promote folding as pharmacological chaperones (Loo et al, 2005; Pedemonte et al, 2005b; Sampson et al, 2011; Wellhauser et al, 2009). Based on this presumption, Kalid et al (2010) used an approach based on the virtual screening (VS) method (omitting the dynamic behaviour of the protein) to identify new modulators of ΔF508-CFTR. In this study, three interdomain cavities on a full-length model of ΔF508-CFTR were used as receptors for molecular docking. Selected molecules were tested at the functional level in various cell types and found to behave either as potentiators or correctors, or even display dual activity. Unfortunately, these molecules were not active in human bronchial epithelial (HBE) cells (Kalid et al, 2010) nor they were tested in CF animal models.

Among the different mechanisms responsible for correcting/potentiating activity, the structural stabilization by binding of small molecules to ΔF508-NBD1 has also been tested. Sampson et al (2011) reported that the corrector VRT-325 and dual activity compound VRT-532 may directly interact with ΔF508-NBD1 and thermally stabilize the protein. A similar observation was made for the RDR1 molecule, which was tested together with 220 correctors previously identified using a cell-based assay (Carlile et al, 2007). Recent findings shown also that VRT-325 exhibits undesirable effect and inhibits the ATPase activity of ΔF508-CFTR by decreasing its affinity for ATP (Kim et al, 2010).

A study by Wieczorek & Zielenkiewicz (2008) revealed the differences in dynamic behaviour between ΔF508-NBD1 and wild type NBD1 (WT-NBD1). Indeed, the NBD1 domain of ΔF508-CFTR exhibits broader conformational freedom than WT-NBD1, which is probably induced by reorganization of the interactions network between residues. Such unique behaviour of mutated NBD1 contributes to the exposure of more hydrophobic regions, which in turn might induce interaction with housekeeping proteins.

On the basis of MD results we suggested in the present work an entirely new mechanism of pharmacological intervention to overcome the dysregulated trafficking of ΔF508-CFTR. We hypothesized that identified small molecules that target the unique conformation of ΔF508-NBD1 may prevent its interaction with protein(s) recognising the mutant as improperly folded and lead to its expression at the plasma membrane. According to this assumption we performed structure-based virtual screening protocol, thus resulted in identification of four new effective correctors of ΔF508-CFTR trafficking; at least two of them might disrupt the interaction between ΔF508-CFTR and keratin 8 (K8).

## RESULTS

### Virtual screening strategy

We have previously shown that the ΔF508 mutation increases the flexibility of NBD1 and, as a consequence, the ΔF508-NBD1 mutant displays a much larger average solvent-accessible surface, which is comprised of hydrophobic residues, than the WT protein (Wieczorek & Zielenkiewicz, 2008). As hypothesized, abnormally large hydrophobic areas on the ΔF508-CFTR protein surface might embody the sites of interaction with housekeeping proteins, leading to premature degradation. In addition, a fraction of ΔF508-NBD1 dynamic trajectory occupies a conformational space unavailable in the WT protein and possesses cavities on the surface, often defined in the literature as ‘druggable’ sites (Nayal & Honig, 2006).

We assumed that binding of small molecules onto these hydrophobic areas could prevent pharmacologically an unwanted situation, protecting ΔF508-CFTR from premature degradation. To address this question, we used a structure-based VS approach to discover chemical compounds with favourable binding characteristics to the ΔF508-NBD1 protein surface.

According to a previous study, the atomic coordinates of the ΔF508-NBD1 frame that is most distinct from WT-NBD1 were adopted as a starting point for molecular docking (MD) (Wieczorek & Zielenkiewicz, 2008). Two wide cavities – pockets 1 and 2 (Supporting Information Fig S1 and Supporting Information Materials and Methods Section) – were then identified around the exposed hydrophobic surface of the ΔF508 domain and treated as two independent receptors for VS.

We decided to screen the National Cancer Institute diversity set I (NCIDS), a relatively modest database consisting of 1990 non-redundant, diverse chemical structures. Molecules were selected according to their unique scaffold as a representative set for over 140,000 available structures from the full NCI database. The NCIDS enabled us to test more extensively the conformational space of the ligands inside the binding pocket in a reasonable amount of time. Each member of the chemical library was docked into potential binding sites using the DOCK program (Moustakas et al, 2006). Commonly used docking algorithms are able to predict the binding conformation of docked ligands inside a receptor with acceptable accuracy; however, though rarely, scoring functions can identify the optimal conformation (Leach et al, 2006). Therefore, we extended the output to the 50 best conformers per molecule (according to internal scoring function) and evaluated them further outside the DOCK program.

In a first step we performed minimization of all selected conformers in rigid surrounding protein using MMFF94 force field. Subsequently, all potential complexes were assessed primarily by value of electrostatic and steric energy contribution calculated by a ‘dock module’ implemented in Sybyl program, and then by various scoring functions selected from the main classes: knowledge-based, molecular force field-based and empirical scoring functions (Leach et al, 2006). On the basis of results from each scoring function, the three best conformations per molecule were saved and subjected to full ligand–receptor minimization and then rescored again, using the selected functions. Finally, instead of using consensus scoring protocols, which have questionable efficiency (Englebienne & Moitessier, 2009), we focused our attention on the top 10 molecules for each scoring function.

After a visual assessment, we identified 12 candidate compounds that were obtained from the NCI as test samples and further evaluated them by biological assays; they were NSC37173, NSC11668, NSC130813, NSC9608, NSC140873, NSC118208, NSC73100, NSC299589, NSC11237, NSC123526, NSC105687 and NSC407882 (Supporting Information Tables S1 and S2).

To test our hypothesis postulating that compounds selected by *in silico* analysis can correct ΔF508-CFTR function, we evaluated the effects of these compounds on several ΔF508-CFTR parameters: protein processing and channel function in three cell lines (HeLa, CF-KM4) and on human bronchial primary epithelial cells from CF patients (CF-HBE) as well as *in vivo* analysis of nasal potential difference in ΔF508/ΔF508 mice.

### Rescue of ΔF508 chloride channel activity by the drugs in HeLa transfected cells

**Figure 1:**
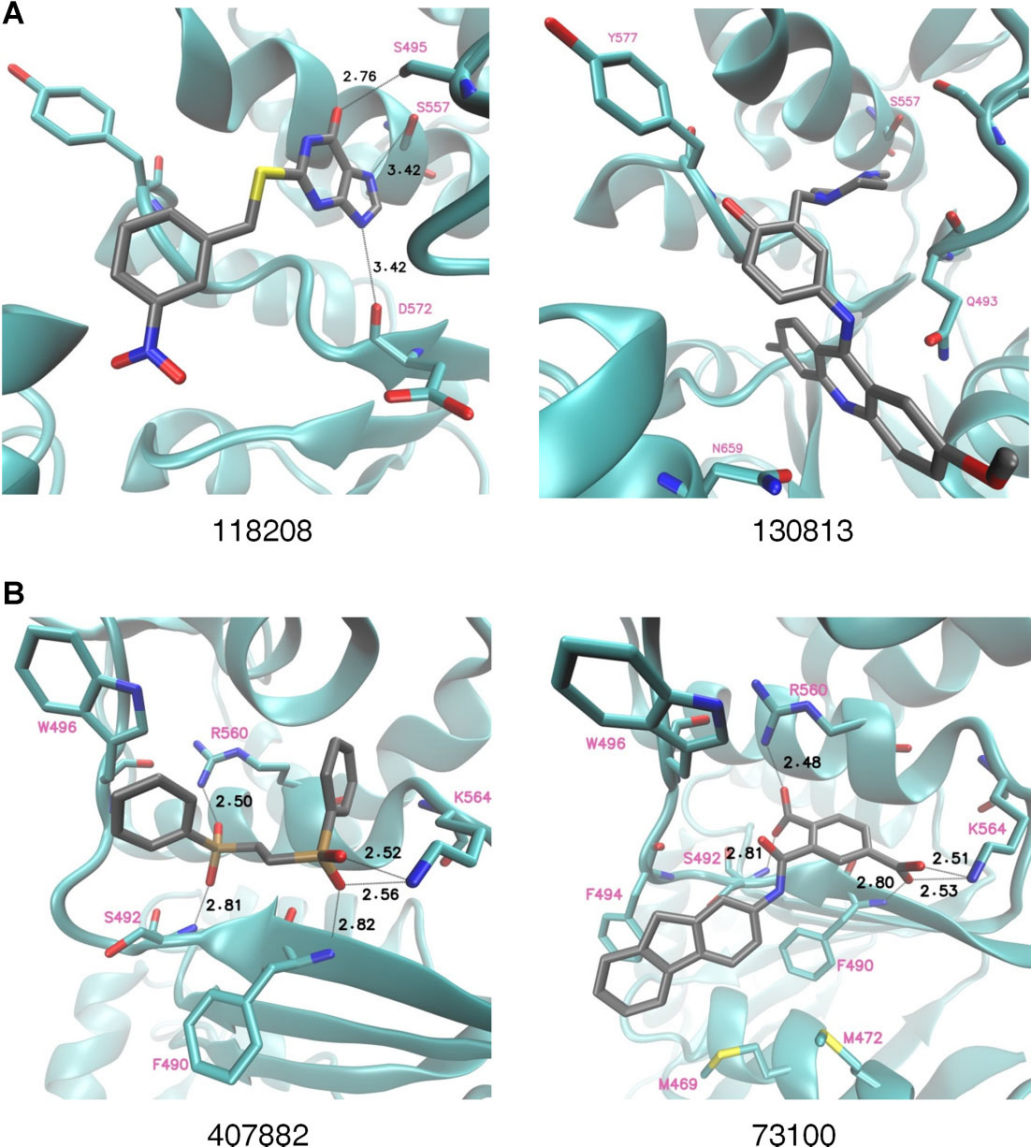
Binding modes for active correctors identified by VS Potential hydrogen bonds are represented by black dashed lines. Distances in Angstroms (Å) are also listed. Essential residues are labelled. Compounds 130813 and 118208 were predicted to bind to pocket 1.The molecules 407882 and 73100 should bind to pocket 2 and such residues as Phe490, Ser492, Arg560 and Lys564 are essential for the formation of a polar interaction with both ligands.

**Figure 2:**
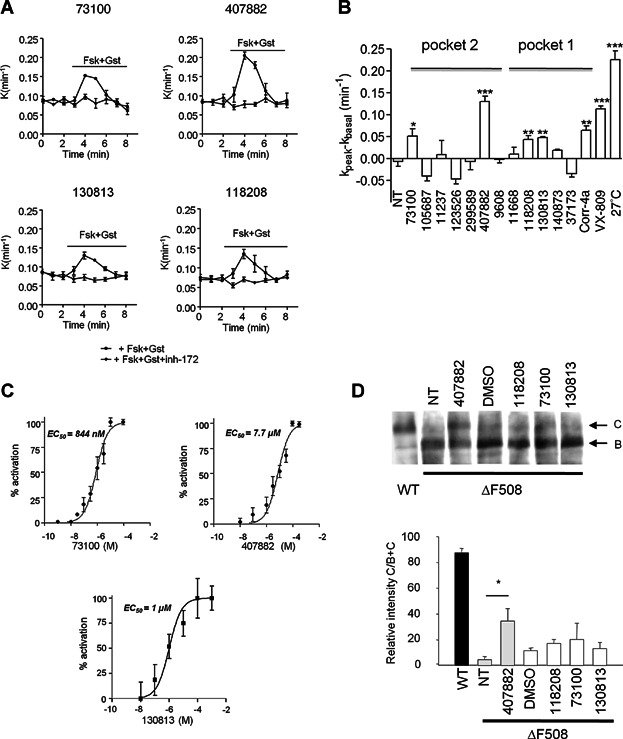
Effect of compounds on iodide efflux and CFTR maturation in ΔF508-CFTR-expressing HeLa cells Representative I^−^ efflux curves obtained in HeLa cells stably transfected with ΔF508-CFTR after treatment for 24 h with 1 µM of the indicated compounds. The CFTR-dependent response was induced by co-treatment with 10 µM forskolin (Fsk) and 30 µM genistein (Gst), as indicated by the horizontal bar above each trace; CFTR-dependent flux was identified by the use of the CFTR inhibitor CFTR_inh_-172 (10 µM).Histogram showing the peak amplitude of Fsk/Gst-dependent I^−^ effluxes in cells treated with the indicated drugs, as shown in A. The values represent the mean + SEM of three independent experiments; *p = 0.04, **p = 0.01 for 118208, *p* = 0.0002 for 130813, *p* = 0.005 for Corr-4a; ***p = 0.01 for 407882, *p* = 0.0005 for VX-809, *p* = 0.0001 for 27°C; Statistics: One-way Anova test followed by Bonferroni post hoc test.EC_50_ for active compounds targeting pocket 2 (407882 and 73100) and pocket 1 (130813). For 118208, EC_50_ could not be precisely determined because the maximum of I^−^ efflux was not reached, even at 100 µM (also shown).Effects of the indicated compounds on CFTR processing. **Upper panel**: representative immunoblots of WT-CFTR and ΔF508-CFTR from HeLa cells treated with 1 µM of the indicated compounds for 24 h in the presence of anti-CFTR monoclonal antibody 24-1. The positions of mature (band C) and immature (band B) CFTR are indicated. Note that WT-CFTR and ΔF508-CFTR are from parallel experiments. **Lower panel**: relative abundance of mature CFTR, expressed as the ratio of band C to bands (C + B). NT, untreated cells. The values represent the mean + SEM of seven independent experiments; *p = 0.005. Statistics: unpaired Student's *t*-test.

To test the effects of drugs on ΔF508-CFTR trafficking and function, we first evaluated I^−^ permeability using a robotic, cell-based macroscopic assay. ΔF508-CFTR-expressing HeLa cells were treated for 24 h with 1 µM candidate correctors and CFTR-dependent radiolabelled iodide (I^−^) efflux was measured after stimulation of cells with 10 µM forskolin (Fsk) and 30 µM genistein (Gst). Assuming that maximal correcting effect is obtained after incubation of cells at 27°C (Denning et al, 1992), the efficiency of correctors was evaluated by comparing I^−^ fluxes with those obtained at 27°C. The efficiency was also compared to that of two reference correctors, Corr-4a and VX-809. Treatments with compounds 130813 and 118208, which target pocket 1, and 73100 and 407882, which target pocket 2 (Fig 1 and Supporting Information Table S1), led to a significant increase in cAMP-stimulated I^−^ efflux (Fig 2A and B), with the most potent molecule being 407882. At this low dose (1 µM), 407882 was less potent than correction at 27°C but twofold more efficient that 10 µM Corr-4a and comparable to 10 µM VX-809. Examples of I^−^ efflux stimulation after treatment with each of the four active compounds are illustrated in Fig 2A. cAMP-stimulated I^−^ efflux was completely inhibited when experiments were performed in the presence of the CFTR channel blocker CFTR_inh_-172.

We further tested the effects of the four compounds on I^−^ efflux using a wide range of concentrations, and determined the EC_50_ for compound 130813, which targets pocket 1, and compounds 407882 and 73100, which target pocket 2, at 1 µM (with 95% confidence interval from 2.1 to 4.9 µM), 7.7 µM (with 95% confidence interval from 5.4 to 1.1 µM) and 844 nM (with 95% confidence interval from 0.6 to 1.2 µM), respectively (Fig 2C). Of note, EC_50_ of VX-809 was reported to be 0.5 µM (Van Goor et al, 2011). On the contrary, the EC_50_ for 118208, which targets pocket 1, was not determined precisely because the maximum I^−^ efflux was not reached, even at 100 µM (data not shown). Inspection of this incomplete concentration–response curve for 118208 suggests an EC_50_ > 100 µM, a value far above those calculated for compounds 130813, 407882 and 73100.

The efficacy of the four compounds as correctors for F508-CFTR trafficking was further evaluated by immunoblot analysis. We assumed that detection of a fully glycosylated protein band (band C of about 170 kDa, mature protein) suggested correct processing of ΔF508-CFTR (Cheng et al, 1991). A representative immunoblot is shown in Fig 2D, upper panel. Anti-CFTR antibodies detect two bands in WT-CFTR cell lysate (WT lane in Fig 2D, upper panel). The diffuse band of approximately 170 kDa corresponds to band C. A second band at about 145 kDa (band B) corresponds to an immature core-glycosylated protein located in the ER. In ΔF508-CFTR-expressing cells, only the immature protein was detectable (ΔF508 NT lane in Fig 2D, upper panel). Band C was clearly detectable in cells treated with 1 µM 407882 compared to untreated cells, whereas the signal at 170 kDa in lysate from cells treated with 1 µM 118208 or 130813 was not significantly different from that of the untreated cells. In addition, C band intensity was very slightly increased in cells treated with 1 µM 73100. The relative abundance of mature CFTR, expressed as the ratio of band C to bands C + B, is shown in Fig 2D, lower panel. Only compound 407882 significantly increased the relative abundance of mature CFTR, in agreement with its most potent activity to increase cAMP-stimulated I^−^ efflux.

To test whether the compounds exhibit potentiator activity independent of their effect on CFTR trafficking, we examined I^−^ efflux in untreated WT-CFTR HeLa cells (Supporting Information Fig S2). Compounds were added along with Fsk, and their effects were compared to that of Fsk alone or Fsk plus Gst (Wellhauser et al, 2009). Unlike Gst, each molecule added with Fsk induced an I^−^ efflux similar to that of forskolin alone, demonstrating that the candidate compounds are not potentiators of WT-CFTR activity.

**Figure 3:**
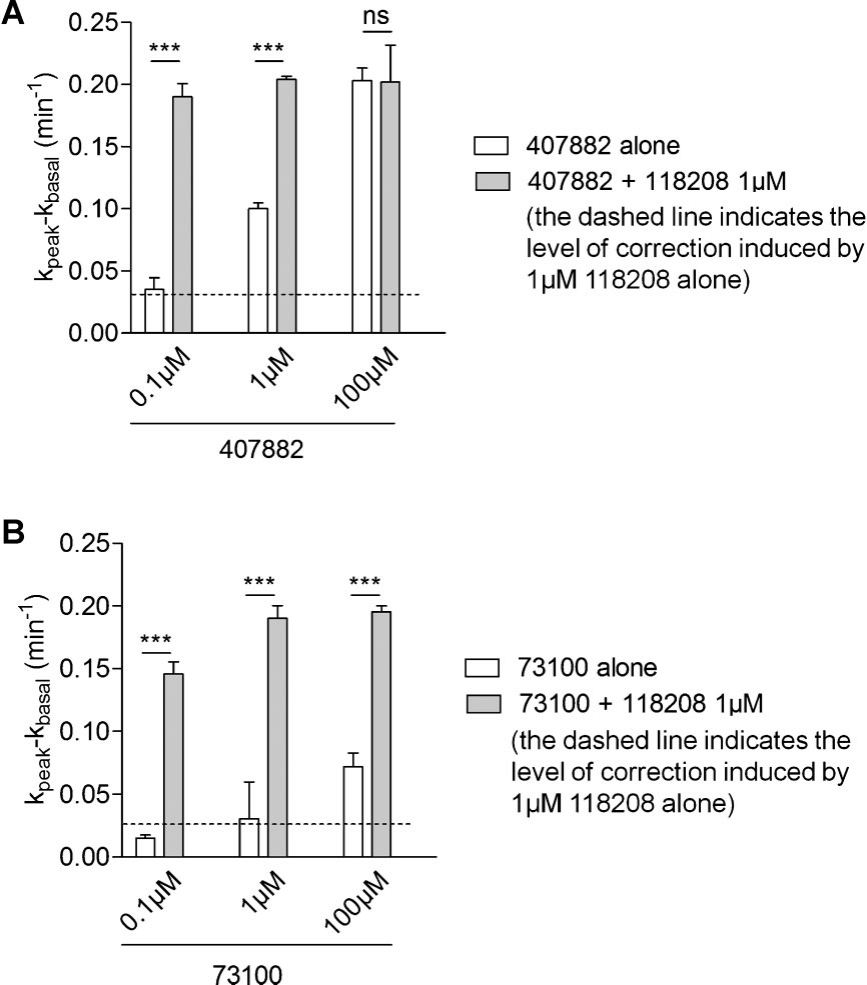
Synergistic effects of active compounds on iodide efflux in ΔF508-CFTR-expressing HeLa cells The CFTR-dependent response was induced by co-treatment with 10 µM Fsk and 30 µM Gst. Histogram of I^−^ efflux for cells treated for 24 h with 118208 and 407882; values represent the mean + SEM of four independent experiments; ***p = 0.0007 for 0.1 µM, *p* = 0.0001 for 1 µM as compared to the amplitudes of individual compounds.Histogram of I^−^ efflux for cells treated for 24 h with 118208 and 73100; values represent the mean + SEM of four independent experiments; ***p = 0.0008 for 0.1 µM, *p* = 0.0006 for 1 µM, *p* = 0.0001 for 100 µM. Statistics: One-way Anova test followed by Bonferroni post hoc test.

If two compounds are able to correct ΔF508-CFTR function by binding to the same protein conformation but at different surface cavities, their effects could be either additive or synergistic. To test for these effects, cells were treated for 24 h with three concentrations of either 407882 or 73100 together with 1 µM 118208. The I^−^ permeability tests (Fig 3) showed that combined treatment with either compound with 118208 results in a more than additive efflux, consistent with a synergistic effect. Of note, the maximal activity that could be measured was achieved when 1 µM 118208 was combined with 0.1 µM 407882 or 1 µM 73100.

**Figure 4:**
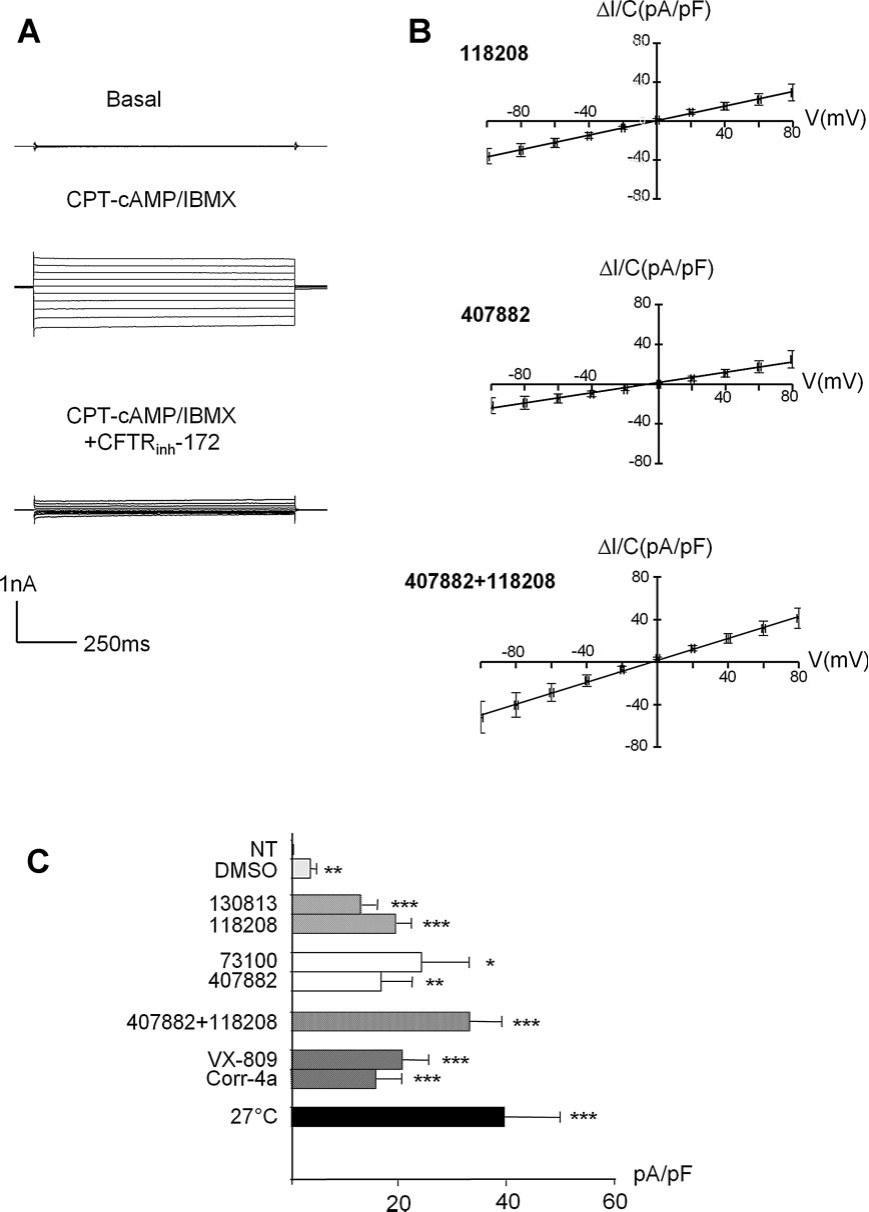
Effect of compounds on whole cell Cl^−^ currents recorded in HeLa cells by patch-clamp experiments Representative current traces recorded by holding the membrane potential at 0 mV and pulsing the voltages in the range −100 to +80 mV at 20 mV steps for a cell treated for 24 h by compound 118208. Current traces recorded: at the basal level (**upper panel**); in the presence of CPT-cAMP/IBMX (**middle panel**); in the presence of CPT-cAMP/IBMX + CFTR_inh_-172 (**lower panel**).Mean CFTR-related current/voltage relationships illustrated for cells treated by compounds 407882, 118208 or 407882 + 118208. Current densities normalized to cell capacitance (pA/pF) were calculated as the differences between current values in the presence of CPT-cAMP/IBMX minus current values after inhibition with 5 µM CFTR_inh_-172.Summary of mean CFTR current amplitudes recorded at −60 mV and normalized to cell capacitance in cells treated for 24 h at 37°C with the indicated compounds or at 27°C as a positive control (means + SEM; *n* = 6–12); *p = 0.05; **p = 0.008 for DMSO, *p* = 0.007 for 407882; ***p = 0.003 for 130813, *p* = 0.00004 for 118208, *p* = 0.0005 for 407882 + 118208, *p* = 0.0007 for VX-809, *p* = 0.003 for Corr-4a, *p* = 0.002 for 27°C, *versus* the corresponding vehicles (water or DMSO). Statistics: unpaired Student's *t*-test. NT, untreated cells.

The activity of each compound was also evaluated using patch-clamp experiments. CFTR-related current density (*I*_ΔF508-CFTR_; pA/pF) is defined as cAMP-stimulated current minus the current recorded after inhibition with 5 µM CFTR_inh_-172. This value is then normalized to cell capacitance. Examples of current traces recorded for one compound (118208) at different voltages from −100 to +80 mV, before and after stimulation with CPT-cAMP/IBMX, and in the presence of CFTR_inh_-172 are illustrated in Fig 4A. *I*/*V* curves representing mean *I*_ΔF508-CFTR_ for cells treated by compounds 118208, 407882 and 118208 + 407882 are shown in Fig 4B. Fig 4C summarizes the mean current amplitudes recorded at −60 mV under different experimental conditions. Treatment for 24 h at low temperature (27°C) or with 10 µM Corr-4A and 10 µM VX-809 served as positive controls, like in I^−^ efflux experiments. Low temperature treatment was the most efficient corrector, increasing current density to near −40 pA/pF, which corresponded to a 90-fold increase as compared to untreated cells cultured at 37°C.

Incubation of cells with either Corr-4A or VX-809 increased current density to −15.8 and −20.8 pA/pF respectively (3.5- and 4.2-fold increase as compared with DMSO treatment alone). Increases in current density comparable to that induced by the two correctors Corr-4A and VX-809 were observed when cells were treated by pocket 1-binding compounds 118208 and 130813 or by pocket 2-binding compounds 407882 and 73100, ranging from −13.1 pA/pF (compound 130813) to −24.2 pA/pF (compound 73100). Of note, the level of current density was not more elevated when cells were treated by compound 407882 as compared with the other compounds, contrary to what was observed during I^−^ flux experiments (Fig 2B). However, in terms of fold increase, current density was increased by near 40-fold by compound 407882 dissolved in water as compared with untreated cells, whereas stimulation ranged from 3.5- to 6.5-fold for the other compounds dissolved in DMSO as compared with vehicle alone. Twenty-four-hour pre-treatment with 1 µM of 118208 plus 1 µM 407882 led to greater *I*_ΔF508-CFTR_ increase than with the compounds alone but this increase did not reach significance as compared to compounds alone.

Treatment of WT-CFTR-expressing HeLa cells with either 407882 or 118208 did not change Δ*I*_CFTR_ (data not shown).

### Correction of CFTR-Cl^−^ conductance in human airway epithelial cells

**Figure 5:**
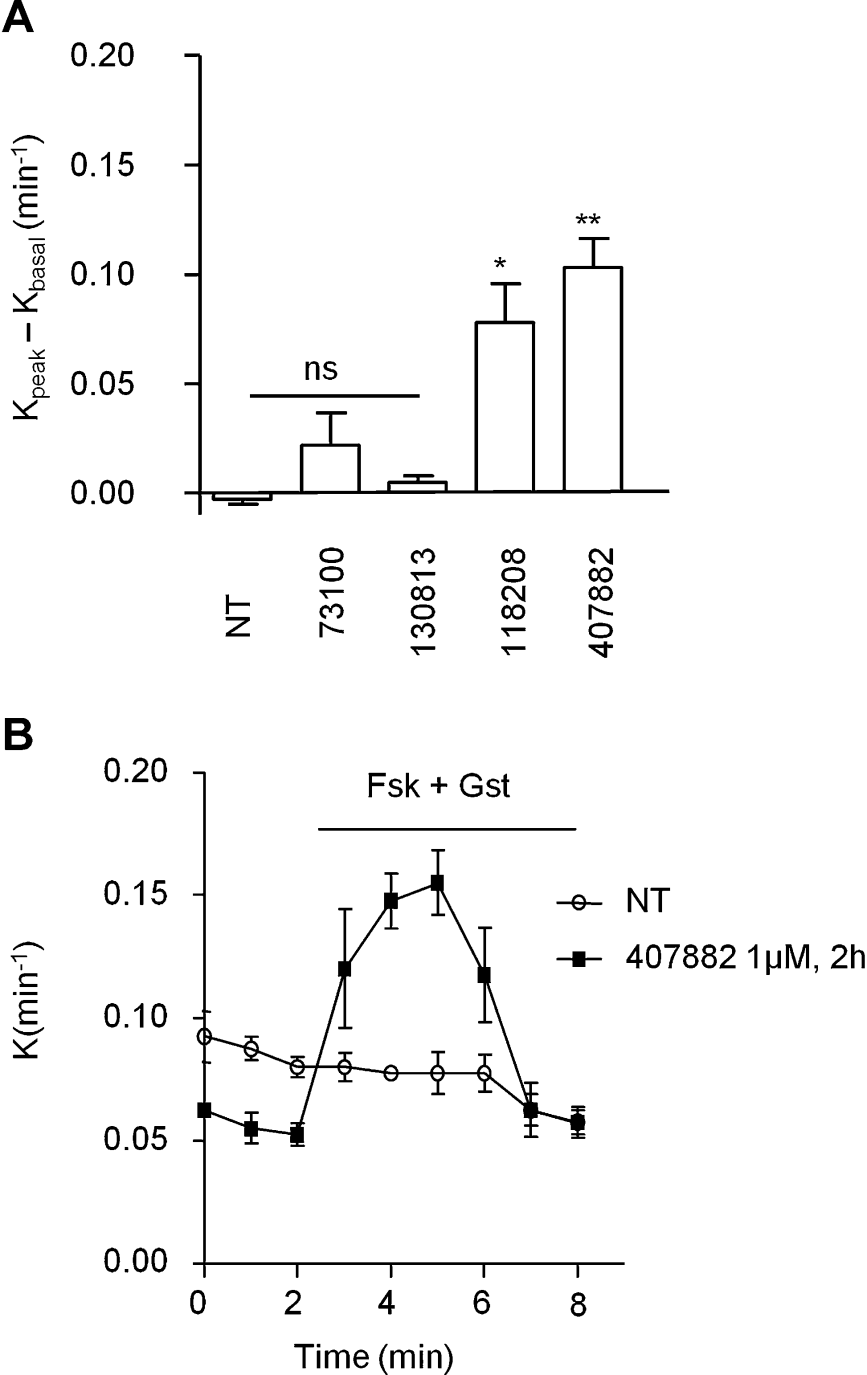
Iodide efflux in CF-KM4 cells Effect of treatment with different compounds (73100, 37173, 118208, 407882) at 1 µM for 2 h. The values represent the mean + SEM of four independent experiments; *p = 0.02, **p = 0.004. Statistics: One-way Anova test followed by Bonferroni post hoc test.Representative I^−^ efflux curves in cells treated for 2 h with 407882 in response to co-treatment with 1 µM Fsk and 30 µM Gst as indicated by the horizontal bar above each trace. NT, untreated cells.

To test if the compounds behaving as correctors in HeLa cells have the same properties in human airway epithelial cells we performed two series of experiments. In the first series, the effects of the four molecules that were active in HeLa cells on CFTR-dependent I^−^ efflux were tested on CF-KM4, an epithelial serous cell line derived from a ΔF508 homozygous CF patient expressing low amounts of endogenous ΔF508-CFTR. Compounds 407882 and 118208 induced significant cAMP-dependent I^−^ efflux in these epithelial cells (Fig 5A; representative I^−^ efflux curve in cells treated for 2 h with 407882 is shown in Fig 5B). However, it must be noted that compounds 130813 and 73100 that modified ΔF508-CFTR function in HeLa cells were not active in this cell line.

**Figure 6:**
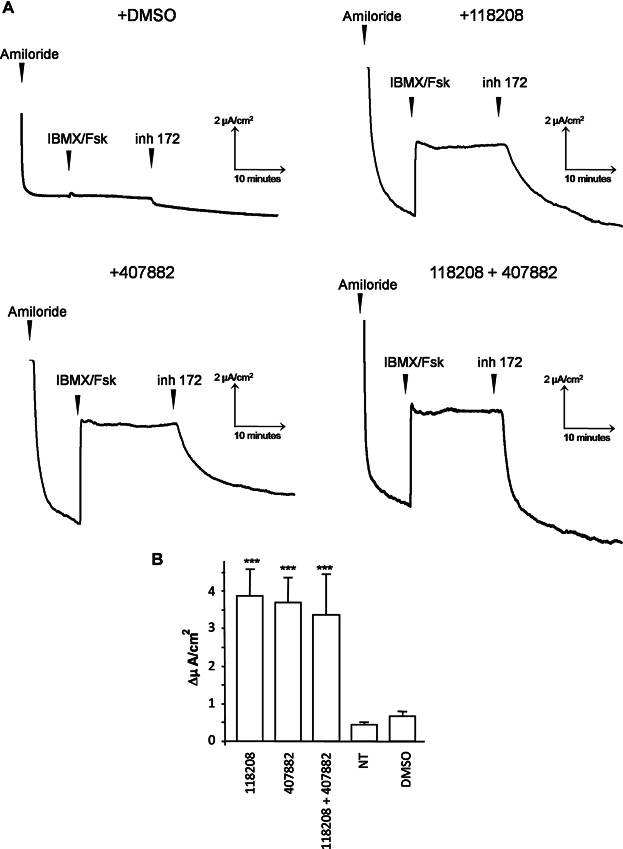
Rescue of ΔF508-CFTR function by compounds 407882 and 118208 in primary airway epithelial cells Representative recordings showing response to 10 µM Fsk/50 µM IBMX and 20 µM CFTR_inh_-172 in cells treated with DMSO (1/50,000), 118208, 407882 or 118208 plus 407882 at 1 µM each for 24 h. Amiloride (100 µM) was first added to the apical side to inhibit Na^+^ absorption occurring through Na^+^ channel (ENaC), followed by addition of Fsk/IBMX (25 and 50 µM).Summary of data for transepithelial Cl^−^ current experiments. CFTR activity was evaluated by measuring the amplitude of the current inhibited by CFTR_inh_-172. Values represent the mean + SEM of four independent experiments; ***p = 3E−08 for 118208, *p* = 8E−09 for 407882, *p* = 1E−05 for 118208 + 407882; Statistics: unpaired Student's *t*-test.

In the second series of experiments, we investigated if the treatment of polarized human bronchial primary epithelial cells from CF patients (CF-HBE) cultured in air–liquid conditions with either 407882 or 118208 (1 µM for 24 h) leads to functional correction of ΔF508-CFTR by short-circuit current experiments (*I*_sc_). Fig 6A shows that in our experimental conditions treatment with 0.0002% DMSO led to almost no changes in *I*_sc_ (upper left panel). Treatment with either 407882 (lower left panel) or 118208 (upper right panel) induced cAMP-dependent Cl^−^ current inhibited by CFTR_inh172_ by 3.9 ± 1.2 and 4.4 ± 1.8 µA/cm^2^, *n* = 4 respectively, suggesting that the observed current was carried by CFTR. Comparatively, in non-CF HBE cells (i.e. cells expressing WT-CFTR), the increase in cAMP-dependent Cl^−^ current inhibited by CFTR_inh172_ was of 24.5 ± 4.5 µA (*n* = 4), indicating that correction was between 15 and 17% of CFTR current. In contrast to the results observed in HeLa cells, in CF-HBE the effect of both compounds applied together was similar to the effect of individual treatments (Fig 6A lower right panel and Fig 6B).

The treatment of HBE cells from two control subjects with either 118208 or 407882 for 24 h did not change the amplitude of cAMP-dependent Cl^−^ current (2.8 and 6.7 µA/cm^2^ before treatment *vs*. 2.5 and 6.9 µA/cm^2^ after treatment).

### Effect of 407882 treatment on nasal potential difference in ΔF508 mice

**Figure 7:**
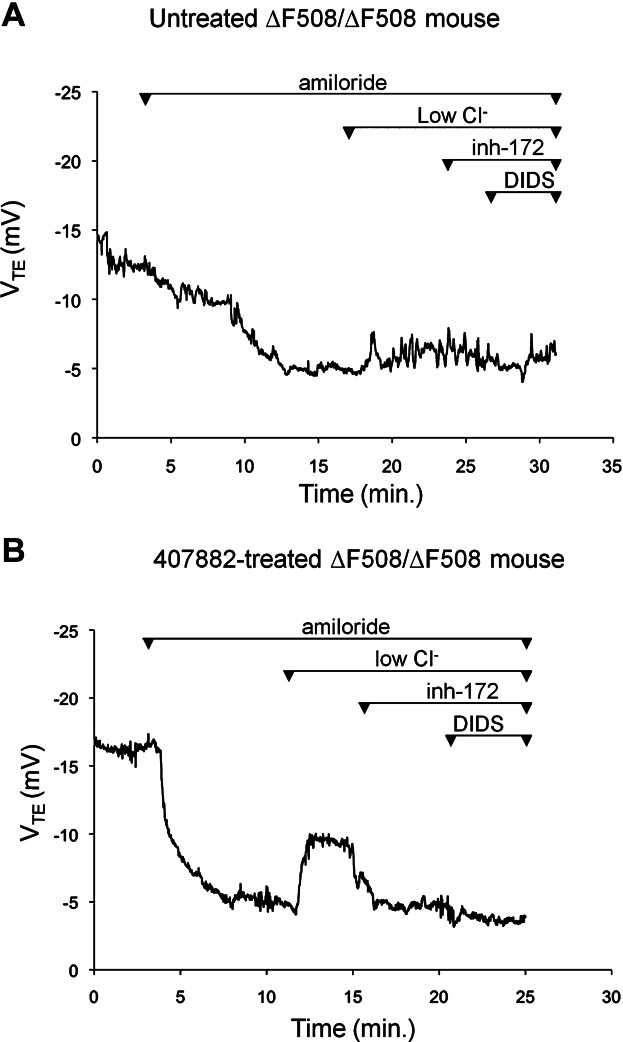
Effect of 407882 on nasal potential difference measurements in ΔF508/ΔF508 mice Representative traces of nasal potential difference measurements in ΔF508/ΔF508 mice before and after treatment with 10 µM 407882 administered in nostril twice within 48 h. 100 µM amiloride-containing solution was applied to inhibit ENaC current, followed by a low Cl^−^ containing solution to unmask Cl^−^ secretion, and subsequently by 5 µM CFTR_inh_-172, and 100 µM of non-CFTR anion transport inhibitor, DIDS. Nasal potential (*V*_TE_) changes recorded 7 days before the first administration of the compound (control).*V*_TE_ recorded 48 h after treatment of the same mouse with compound 407882.

We tested on a ΔF508/ΔF508 mouse model whether treatment of nasal epithelium with one of the pocket 2 molecules leads to correction of CFTR-related Cl^−^ secretion by measuring nasal potential difference changes (Δ*V*_TE_) (Sermet-Gaudelus et al, 2010). Among these molecules we chose 407882 because of its water solubility, and used the following protocol: at Day 0 *V*_TE_ was measured; next week, at Days 7 and 8, 20 µl of 10 µM 407882 was instilled into the nostrils of mice and *V*_TE_ was measured again at Day 10. We observed that treatment of nasal epithelium with 407882 (according to the protocol described in Materials and Methods Section) resulted in CFTR_inh_172-senstive hyperpolarization of Δ*V*_TE_ compatible with activation of the CFTR-Cl^−^ channel (4.8 ± 1.8 mV in four tested mice *vs*. 0.2 ± 1.3 mV in three untreated mice; *p* = 0.006 using unpaired Student's *t*-test). It must be noted that no inh172-sensitive hyperpolarization of Δ*V*_TE_ was detected in control mice (Fig 7). By comparison, Δ*V*_TE_ in response to low Cl^−^ perfusion solution in WT mice was −10.3 ± 4.5 mV, *n* = 8.

### Inhibition of ΔF508-CFTR-keratin 8 interaction by compounds 407882 and 118208

**Figure 8:**
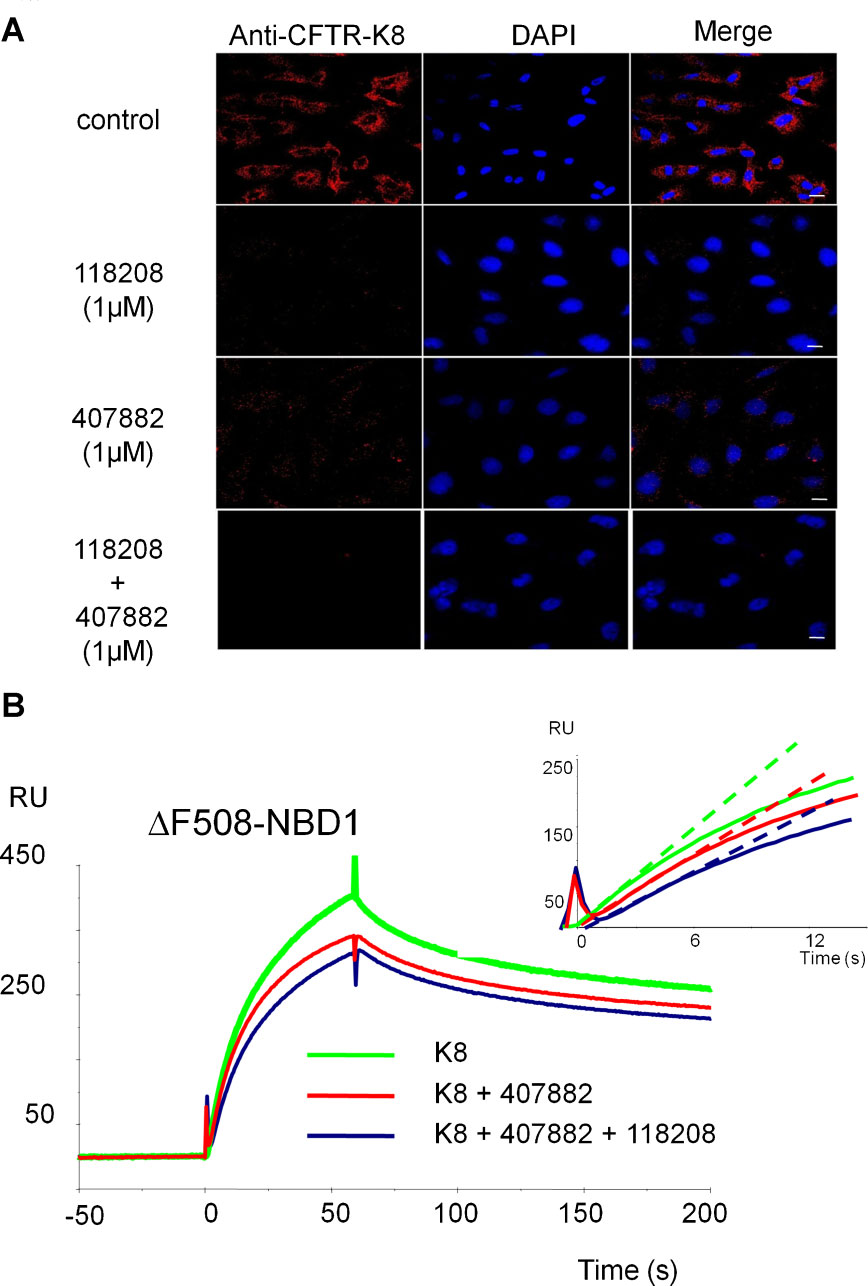
Effect of 407882 and 118208 on interaction between K8 and CFTR in ΔF508-CFTR-expressing HeLa cells. Proximity ligation assay (PLA) of K8 and ΔF508-CFTR in transfected HeLa cells. Red dots correspond to K8/ΔF508-CFTR interaction (proximity <40 nm), and blue staining to nuclei (DAPI). Control: untreated cells. Results are representative of at least three independent experiments.SPR analysis of K8 binding to human ΔF508-NBD1 (2900 RU = 2900 pg mm^−2^) in the absence (green curve) and presence (red curve) of 180 µM 407882 and/or (blue curve 407882 plus 118208 compounds). Insert: expanded curves of initial RU change showing the changes in initial slopes (dashed lines).

We have shown in a previous study that disruption of interaction between the intermediate filament protein keratin 8 and ΔF508-CFTR leads to functional correction of the mutated protein (Colas et al, 2012). To test whether the compounds investigated in the current study interrupt this interaction, we performed two series of experiments: (i) a proximity ligation assay on HeLa cells expressing ΔF508-CFTR, and (ii) a surface plasmon resonance assay (SPR). Fig 8A has been shown a dramatic decrease in interaction, indicated by fluorescent red spots, after treating cells with 1 µM of either 407882 or 118208, or of both compounds (each dot reflects a proximity of <40 nm between a K8 and ΔF508-CFTR pair). Fig 8B is shown representative traces of K8 binding to ΔF508-NBD1 in the presence and absence of 407882 and 407882 + 118208. Measurement of the maximum amounts of bound K8 (see Supporting Information Materials and Methods Section) after K8 addition indicates that either 407882 or 407882 + 118208 diminished by 15–20% the K8 association with ΔF508-NBD1. Evaluation of the initial association slope K8 (ΔRU/s) showed that the association rate diminishes by 10–22% in the presence of 407882, and ∼40% in the presence of both compounds (two independent experiments).

These data suggest that 407882 and 118208 perturbed K8 and ΔF508-CFTR interactions, what might imply an overlapping between sites of action for K8 and both correctors.

### Effect of compounds 407882 and 118208 on ΔF508-NBD1 and WT-NBD1 structure

To test if the two compounds have a direct influence on NBD1 structure, we have applied hydrogen-deuterium exchange reaction coupled with mass spectrometry (HDex-MS). According to the results from MD simulations the ΔF508-NBD1 structure conformation used for virtual screening (a docking frame) protocol was unobservable in the WT-NBD1 trajectories (Wieczorek & Zielenkiewicz, 2008). However, the analysis of WT-NBD1 trajectory only in the regions of two binding pockets has shown, that congenial conformations of residues forming pocket 2 in ‘the docking frame’ could be observed (RMSD ∼1.5 Å, Supporting Information Fig S3), whereas conformation of residues forming pocket 1 in the ‘docking frame’ was unachievable for WT-NBD1 during all MD simulation time (Supporting Information Fig S3). This suggests, that at least 118208 compound should bind to ΔF508-NBD1 domain but not to the WT. To verify this *in silico* observation, HDex-MS experiments were performed with both WT and ΔF508-NBD1 proteins.

The global patterns of relative deuterium uptake in the experiments in the presence and absence of compounds after 10 s of reaction for both proteins were similar but with noticeable changes (Supporting Information Fig S4). A careful analysis (Kreyszig, 1993; Lazar & Schwartz, 1967) revealed that some differences in HDex reaction rates (NBD1s in the presence of compounds *vs*. NBD1s without compounds) were outside of *X* ± *σ* and therefore statistically significant (Supporting Information Figs S5 and S6). The average changes in deuterium uptake after addition the 118208 molecule were 2.3% for WT-NBD1 and 3.7% for ΔF508-NBD1, whereas 407882 compound induced similar changes in both cases, 5.3 and 5.4% for WT-NBD1 and ΔF508-NBD1 respectively, suggesting a slight global destabilising effect of both compounds on ΔF508 and WT-NBD1 structures.

**Figure 9:**
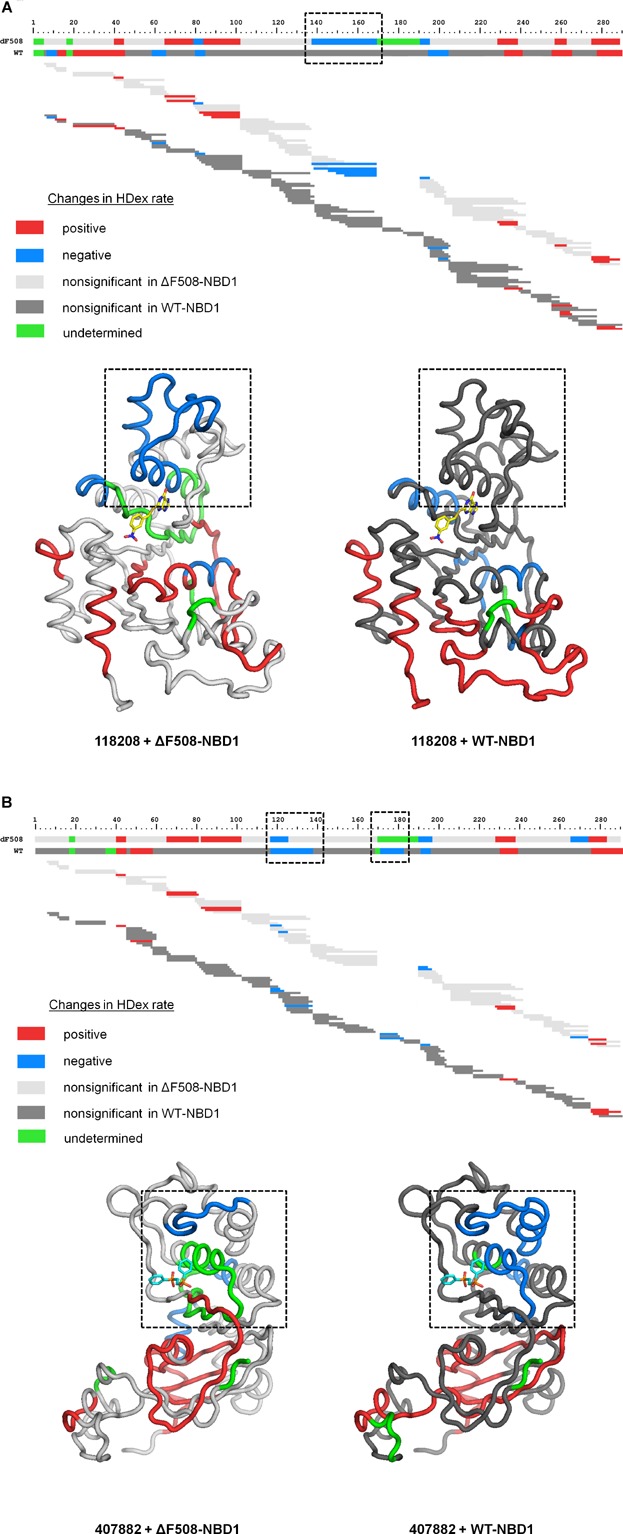
Effect of compounds 118208 and 407882 on ΔF508 and WT-NBD1 structures tested by HDex-MS experiment The coverage of ΔF508 and WT-NBD1 sequences by peptic peptides has been shown on (upper panels). The peptides have also been mapped onto the ΔF508-NBD1 structure – ‘docking frame’ (lower panels), and coincidences between localization of binding pockets for each molecule and peptides with diminished hydrogen-deuterium exchange rate have been indicated by dashed lines. Sequence coverage by peptic peptides was 94.5% for ΔF508-NBD1 and 97.5%, for WT-NBD1. 118208.407882.

All peptic peptides with significant changes in HDex rate for both ΔF508 and WT domains after addition of tested compounds have been mapped onto ΔF508-NBD1 ‘docking frame’ structure (Fig 9A and B).

For ΔF508-NBD1 peptides for which HDex rate changes were registered upon presence of 118208 compound, seven had either diminished (five peptides) or not sufficiently increased (two peptides; Supporting Information Table S3 and Supporting Information Fig S5A and B). Four out of these seven peptides cover the region from L526 to L558 and are partly overlapped with pocket 1 (Fig 9A). More detailed analysis of deuterium uptake in peptides from this region of NBD1 indicates that their relative decrease in HDex rates is actually limited to the smaller region N538 to L558 which is in pocket 1 (Supporting Information Fig S6A and B).

Regarding 407882 molecule, among peptic peptides with significant changes in HDex rate detected for ΔF508-NBD1, only four had diminished and one had not sufficiently increased in HDex to be comprised within range of 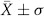 (Supporting Information Table S3 and Supporting Information Fig S5C and D). Two out of those five identified peptides encompass the residues participate in the region of pocket 2 (Fig 9B). For experiment with WT-NBD1 in the presence of 407882 compound, surprisingly we have identified 4 peptides covered the region of pocket 2 with significant diminished HDex rate: N505-G509, N505-S511, A559-D567 and A559-L570 (Fig 9B, Supporting Information Table S4 and Supporting Information Fig S6C and D). Additionally, the peptide D579-E583 exhibits diminished HDex rates after incubations ΔF508-NBD1 with either of compounds as well as WT-NBD1 with 407882 compound (Supporting Information Tables S3 and S4). This effect is probably not specific allosteric reaction of structural changes.

Altogether these results suggest the 118208 compound affects region of pocket 1 only in ΔF508-NBD1, whereas 407882 affects region of pocket 2 in ΔF508 and WT-NBD1, what in fact is convergent with MD simulation results.

### Binding areas *versus* full CFTR model

Two binding areas for virtual screening protocols have been selected according to the results from ΔF508-NBD1 molecular dynamic simulation only (Wieczorek & Zielenkiewicz, 2008). Thus it was of interest to localized those binding places onto the structural model of the full CFTR protein. The ΔF508-NBD1 docking frame has been superimposed onto CFTR structural coordinates prepared by Mornon et al (2008). As has been shown on Supporting Information Fig S7, the binding region for 407882 and 73100 compounds (pocket 2) is occupied by ICL4 loop of the TMD2. The binding area for 118208 and 130813 (pocket 1) has been localized on the deep interaction interface between NBD1 and NBD2, and in the CFTR model is occupied by two loops from NBD2 domain.

## DISCUSSION

In this work we utilize virtual screening strategy together with the results from molecular dynamic simulation (Wieczorek & Zielenkiewicz, 2008) to discover very effective compounds, that are able to overcome trafficking defect of ΔF508-CFTR protein leading to correction of CFTR function in ΔF508/ΔF508 CF human cells and mice. All four molecules represent different chemical classes (Fig 1 and Supporting Information Table S1), which are novel in the field of ΔF508-CFTR pharmacology and might provide a novel mechanism of action.

The efficacy of the compounds was confirmed using several assays based on the measurement of CFTR_inh_-172-sensitive I^−^ efflux, CFTR_inh_-172 sensitive chloride current and ΔF508-CFTR processing in different cell types. The EC_50_ of the four described correctors ranged between 0.8 and >100 µM, the active concentrations for all compounds being 1 µM. This indicates that the identified molecules lie within the same correction range as VX-809, considered as the best corrector described to date (currently being subjected to a clinical trial; http://clinicaltrials.gov), and substantially more effective than VRT-325 (Van Goor et al, 2006) and RDR1 (Sampson et al, 2011). Notably, the effect of 1 µM 407882 on HeLa cells was as potent as 10 µM of VX-809.

Additive and/or synergistic correction was observed in ΔF508-CFTR-expressing HeLa cells treated with two compounds, either 407882 plus 118208 or 73100 plus 407882, reaching the correction amplitude observed in cells cultured at 27°C. In CF HBE primary cultured cells treated with 1 µM of 407882, Cl^−^ transport reached 15% of non-CF HBE cells. This represents the same level of Cl^−^ transport correction as with 3 µM VX-809 in cultured HBE cells, as previously described (Van Goor et al, 2011). Importantly, 407882 is effective *in vivo* since it corrects CFTR function in ΔF508/ΔF508 mice. The fact that correction by 407882 was observed in all tested cell types is an important finding, while it has been reported that correcting effect is cell type dependent (Pedemonte et al, 2005b). We even anticipate that the cell background effect of our correctors may be less significant due to their potential binding to ΔF508-CFTR surface rather than to interaction with proteins engaged in degradation pathways, which can be specific for each cell type.

The most promising molecule, 407882, contains two phenylphosphinic acid moieties, which occupy pocket 2 (Fig 1B). The highly polar hydroxyphosphoryl groups create interactions with protein residues such as Lys564, Arg560 and Ser492, whereas the two phenyl rings are bound to the more hydrophobic portions of pocket 2. The second compound that was also predicted to bind to pocket 2, 73100, is chemically distinct from the first one, as it comprises terephthalic acid and fluorene moieties. Nevertheless, both 407882 and 73100 share the same pharmacophore. The two carboxyl groups from the tetrapthalic moiety of 73100 perfectly overlap with the two hydroxyphosphoryl groups of 407882, thus creating electrostatic interactions with the same protein residues (Fig 1B), whereas the fluorene moiety is positioned in the hydrophobic cleft of pocket 2 formed by Phe494, Phe490, Met469 and Met472. Among the compounds predicted to bind to pocket 1, 118208 represents a hypoxanthine derivative substituted with (3-nitrophenyl)methylsulfanyl group in position 2 of a purine-like ring. The hypoxanthine part fits perfectly into the deep cavity on ΔF508-NBD1 surface and may create three H-bonds with protein residues Ser495, Ser557 and Asp572 (Fig 1A). The phenyl ring is positioned in the neighbourhood of Tyr577.

Compound 130813 consists of an acridine ring, which has recently been identified by Sondo et al as a corrector of ΔF508-CFTR trafficking (Sondo et al, 2011). Since these authors suggested that 9-aminoacridine acts as a proteostasis regulator, our hypothesis in case of 130813 needs to be interpreted with caution. Nevertheless, the structure of 130813 is much more arborescent and the 9-aminoacridine ring is substituted with a chloride atom, a methoxyl group and a very bulky 3-hydroxy[(4-methylpiperazin-1-yl)methyl]phenyl moiety. The binding mode of 130813 to pocket 1 significantly differs from that of 118208; however, the phenyl ring occupies a similar position in both cases.

The concept of drug binding sites on the mutated NBD1 has already been evoked with regard to potentiators by Moran et al (2005) and recently proved experimentaly by differential scaning fluorimetry for RDR1 corrector (Sampson et al, 2011). In our studies we used HDex-MS technique, to check whether our computationally predicted molecules might in fact specifically interact with ΔF508-NBD1. Contrary to expectations, we observed rather a slight global destabilization of NBD1 proteins by 118208 and 407882 compound. However this destabilization was prevented in regions overlapped with *in silico* predicted pockets 1 and 2. This observation might suggest direct interactions with ΔF508-NBD1.

Interestingly, neighbourhood of binding region for 407882 has been already indicated by Mornon as potential target for pharmacological intervention by small molecule (Mornon et al, 2008). According to the authors such compound should form stabilize interaction between NBD1 and ICL4, which is suspected to be altered in ΔF508-CFTR and causes folding defect (Mendoza et al, 2012; Rabeh et al, 2012). It also has been used for the first virtual screening strategy to identified new modulators of ΔF508-CFTR (Kalid et al, 2010) and were indicated as putative binding place for VX-809 (He et al, 2013). In both cases, *in silico* studies were performed on full length CFTR structural model and binding pockets consisting of NBD1 and ICL4 residues. In contrast to previous studies the binding pocket for 407882 is fully localized onto the NBD1 domain, and molecule perfectly occupied place where ICL4 loop should reside (Supporting Information Fig S7).

In the studies presented by Kalid et al, three putative cavities on the full-length CFTR structural model have been defined as independent receptors for VS (Kalid et al, 2010). The most important difference between this study and ours was the conceptual approach. Firstly, they tried to identify molecules that would stabilize the protein by potentially affecting either the folding yield or the surface stability. Thus, all putative binding places were identified on the full CFTR protein and were localized on inter-domain interfaces like NBD1-NBD2, NBD1-ICL4 and NBD1-NBD2-ICL1:2:4. Secondly, authors omitted an exhaustive testing of dynamic behaviour of ΔF508-CFTR, what was a crucial aspect in our VS protocol. Especially in the case of binding pocket 1, which was absent in the ΔF508-NBD1 crystal structure.

Among the most efficient ΔF508-CFTR correctors described to date VX-809, Corr-4a and VRT-325 are also suspected to directly target the structure of mutated protein, however the putative site of action for all of them remain unknown (He et al, 2013; Kim et al, 2010; Wang et al, 2007), and for the last two some results suggested even more general mechanism involved in protein processing (Van Goor et al, 2011). Commonly accepted mechanisms by which molecules induce protein trafficking to the plasma membrane is stabilization of the protein structure and promotion of proper folding.

However for our study, we have to keep in mind that an ‘abnormal’ conformation was targeted, which might suggest a different way of action. Thus we postulate the alternative concept of correction mechanism for our molecules, which has been built on previous results from MD studies on WT-NBD1 and ΔF508-NBD1 domains (Wieczorek & Zielenkiewicz, 2008). In this context, we believe that we have identified molecules that probably inhibit interactions between ΔF508-CFTR and housekeeping protein, rather than stabilize a mutated domain that is very distinct from the native one. Moreover, our results rule out the stabilization of NBD1 as a mechanism of action of new correctors, thus HDex-MS experiments have shown only slightly stabilization of binding pockets regions, whereas over all structure of ΔF508-NBD1 is rather destabilized in presence of our compounds. Previously, we have shown that disruption of interaction between ΔF508-CFTR and keratin 8 leads to restoration functional ΔF508-CFTR. We proposed site of K8-ΔF508-CFTR interaction as novel therapeutic target for ΔF508/ΔF508 CF patients (Colas et al, 2012). The results of proximity DNA ligation and SPR experiments are in favour of this hypothesis (Fig 8).

In conclusion, the chemical compounds identified by us as correctors of ΔF508-CFTR are very promising as potential therapeutic agents. A correction level between 10% and 30% of the WT-CFTR activity has been estimated as the threshold required to ameliorate the symptoms of CF (McKone et al, 2003; Zhang et al, 2009). In our study, this correction threshold is obtained even by treatment with a single molecule, something that has been attainable with VX-809 (Van Goor et al, 2011). Notably, the fact that our most potent molecule, 407882, is water-soluble represents a favourable characteristic for drug administration.

## MATERIALS AND METHODS

### Study approval

All animal protocols used in the present study were approved by the Institutional Animal Care and Use Committees of the INSERM.

### Virtual screening

Detailed information about preparation of NCIDS database and receptors using SYBYL 7.3 as well as full description of VS procedure using Dock 6.1 (Moustakas et al, 2006) are provided in Supporting Information Materials and Methods Section.

### Reagents and antibodies, cell culture, immunoblot experiments, transepithelial Cl^−^ current measurements and hydrogen deuterium exchange MS

All reagents, antibodies, detailed information on cell culture, transepithelial ion transport and hydrogen deuterium exchange MS are provided in Supporting Information Materials and Methods Section.

### Iodide efflux experiments

CFTR chloride channel activity was assayed by measuring iodide (^125^I) efflux from transfected HeLa cells as described previously (Marivingt-Mounir et al, 2004). Detailed information is provided in Supporting Information Materials and Methods Section.

### Whole cell patch clamp recordings

The technique for patch clamp recordings in the whole cell configuration has been described elsewhere (Hinzpeter et al, 2006; Tanguy et al, 2008). Detailed information is provided in Supporting Information Materials and Methods Section.

### Nasal potential difference (NPD) measurements

The method for nasal potential measurement was adapted from the technique developed for young children (Sermet-Gaudelus et al, 2010). Detailed information is provided in Supporting Information Materials and Methods Section.

### Proximity ligation assay

Cells were fixed with cold acetone and analysed using the Duolink™ kit (Eurogentec, Angers, France) according to manufacturer's instructions. Detailed information is provided in Supporting Information Materials and Methods Section.

### Surface plasmon resonance

Protein–ligand interactions were studied in real time using a SPR Biacore 2000 system and CM5 sensor chips (GE Healthcare). NBD1 (WT and ΔF508) were covalently immobilized via primary amino groups on the sensor chip surface, at 20°C, as described by Colas et al (2012). Other details are in Supporting Information Materials and Methods Section.

**The paper explained**

PROBLEM:

Cystic fibrosis (CF) is a fatal autosomal recessive genetic disorder caused by mutations in the *CFTR* gene, which encodes CF transmembrane conductance regulator (CFTR), a protein with Cl^−^ channel functions. Deletion of Phe508 (ΔF508) in the first nucleotide binding domain (NBD1) of CFTR is the most common mutation associated with CF. The ΔF508-CFTR is recognized as improperly folded and targeted for proteasomal degradation. Thus, a significant decrease in functional CFTR expression occurs at the apical plasma membrane that leads to abnormal Cl^−^ transport across numerous epithelia and is responsible for a severe form of CF.

RESULTS:

Here we apply a virtual screening strategy together with the results from molecular dynamics simulation to discover four very effective compounds (correctors), that are able to overcome the defect of ΔF508-CFTR and leading to correction of CFTR function in various cell types including primary culture of human bronchial CF cells and the nasal epithelium of homozygous ΔF508-CFTR mice. Using different functional assays we demonstrate that the newly identified compounds induce functional expression of ΔF508-CFTR within the same correction range as VX-809, considered as the best corrector described to date (and currently being subjected to a clinical trial). We also propose a new mechanism of action for the identified molecules, possible based on the inhibition of the ΔF508-CFTR and keratin 8 interactions, as supported by proximity DNA ligation and surface plasmon resonance experiments.

IMPACT:

These compounds are among the most potent correctors of the ΔF508-CFTR trafficking defect known to date and appear very promising as potential therapeutic agents.

## Supplementary material

As a service to our authors and readers, this journal provides supporting information supplied by the authors. Such materials are peer reviewed and may be re-organized for online delivery, but are not copy-edited or typeset. Technical support issues arising from supporting information (other than missing files) should be addressed to the authors.

**Figure S1. Two binding pockets on the ΔF508-NBD1 surface.**

**Figure S2. A. Representative iodide efflux curves obtained in HeLa cells stably transfected with WT-CFTR.**

**Figure S3. The local analysis of WT-NBD1 trajectory only in the areas of two binding regions.**

**Figure S4**. **A–D. Histogram of deuterium uptakes in ΔF508-NBD1 in the presence of compounds 118208 or 407882.**

**Figure S5. A–D. Differences in deuterium uptake between ΔF508-NBD1 incubated with 118208 (A)/(B) or 4078823 (C)/(D) compounds %*D***_**drug**_ **− %*D***_**control**_ **(% deuterium**_**drug**_ **− % deuterium**_**control**_**).**

**Figure S6. A–D. Differences in deuterium uptake between WT-NBD1 incubated with 118208 (A)/(B) or 4078823 (C)/(D) compounds %*D***_**drug**_ **− %*D***_**control**_ **(%deuterium**_**drug**_ **− %deuterium**_**control**_**).**

**Figure S7. The full-length CFTR model coordinates have been adopted from Mornon et al (2008).**

**Table S1. Compounds selected by virtual screening procedure for experimental tests.**

**Table S2. Virtual screening results from each scoring function for selected ligands.**

**Table S3. Peptides with diminished HDex rates in the presence of correcting compounds.**

**Table S4. Peptides of WT-NBD1 with diminished HDex rates in the presence of correcting compounds.**

**Table S5. DynamX criteria for filtering the list of ΔF508-NBD1 peptides from PLGS program.**

**Table S6. Parameters for processing MS spectras by DynamX program.**
