# Supplementary material for: Discovery of novel potent ΔF508-CFTR correctors that target the nucleotide binding domain
Source: EMBO Mol Med. 2013 Aug 27;5(10):1484–501. doi: 10.1002/emmm.201302699 (PMC3799575; doi:10.1002/emmm.201302699)
Supplement: Supplementary file 1 [file emmm0005-1484-SD1.pdf]

EMBO Molecular Medicine

Discovery of novel potent F508-CFTR correctors that target the nucleotide binding domain

Norbert Odolczyk, Janine Fritsch, Caroline Norez, Nathalie Servel, Melanie da Cunha, Sara Bitam, Anna Kupniewska, Ludovic Wiszniewski, Julien Colas, Krzysztof Tarnowski, Danielle Tondelier, Ariel Roldan, Emilie Saussereau, Patricia Melin-Heschel, Grzegorz Wieczorek, Gergely Lukacs, Michal Dadlez, Grazyna Faure, Harald Herrmann, Mario Ollero, Frédéric Becq, Piotr Zielenkiewicz and Aleksander Edelman

Corresponding author: Piotr Zielenkiewicz, Institute of Biochemistry and Biophysics, Polish Academy of Sciences and Aleksander Edelman, INSERM

Review timeline:

Submission date:	19 March 2013
Editorial Decision:	02 May 2013
Revision received:	02 July 2013
Editorial Decision:	08 July 2013
Revision received:	18 July 2013
Accepted:	19 July 2013

Transaction Report:

(Note: With the exception of the correction of typographical or spelling errors that could be a source of ambiguity, letters and reports are not edited. The original formatting of letters and referee reports may not be reflected in this compilation.)

Editor: Roberto Buccione

1st Editorial Decision 02 May 2013

Thank you for the submission of your manuscript to EMBO Molecular Medicine. We have now heard back from the three Reviewers whom we asked to evaluate your manuscript.

You will see that all three Reviewers are generally supportive of you work although Reviewer 2, in particular, expresses some concerns that prevent us from considering publication at this time.

Reviewer 2 points to a number of experimental issues that require your action. S/he is especially concerned that key experiments lack sufficient controls and that the pharmacological conclusions require additional work and clarification. S/he also notes that direct evidence of the effect of the active compound on the keratin 8-CFTRinteractions is required. I will not dwell into much detail, as the many action points are detailed, self-explanatory and well taken.

Reviewer 3 would like you to add controls (wild-type CFTR) for the deltaF506-CFTR rescue experiments depicted in Figure 6.

Considered all the above, while publication of the paper cannot be considered at this stage, we would be pleased to consider revised submission, with the understanding that the Reviewers'

concerns must be fully addressed, with additional experimental data where appropriate and that acceptance of the manuscript will entail a second round of review.

Please note that it is EMBO Molecular Medicine policy to allow a single round of revision only and that, therefore, acceptance or rejection of the manuscript will depend on the completeness of your responses included in the next, final version of the manuscript.

As you know, EMBO Molecular Medicine has a "scooping protection" policy, whereby similar findings that are published by others during review or revision are not a criterion for rejection. However, I do ask you to get in touch with us after three months if you have not completed your revision, to update us on the status. Please also contact us as soon as possible if similar work is published elsewhere.

I look forward to seeing a revised form of your manuscript as soon as possible.

***** Reviewer's comments *****

Referee #1 (Comments on Novelty/Model System):

This manuscripts presents data obtained with a state-of-the-art combination of computational, biophysical and biochemical. Results are highly relevant for the possible treatment of cystic fibrosis patients.

Referee #1 (General Remarks):

This work represent a great effort to face a problem beginning with a theoretical approach, followed by a series of biochemical, and functional experiments in cell, tissue a whole-animal models, concluding with a molecular experiments that provides an explanation that are perfectly coherent with the starting molecular models. The main aim of the work, searching for substances to correct CFTR defects, is well accomplished. The whole procedure could be used for searching new, "drugable", compounds for the cystic fibrosis treatment.
The manuscript describes a long series of sophisticated experiments in a perfectly logic sequence. A nonspecialist may have difficulties to follow all experiments, but the conclusion are well explained to put in evidence the nature and aim of each experiment.

Referee #2 (Comments on Novelty/Model System):

This is a multidisciplinary study that used complementary approaches and technologies to discover and characterize the activity of deltaF508-CFTR correctors with the aim of identifying novel therapeutics for the treatment of cystic fibrosis. In this regard, technical quality, novelty and medical impact are high. The model system used is also appropriate and no ethical concerns are raised.

Referee #2 (General Remarks):

The manuscript by Odolczyk et al. describes the discovery of novel corrector molecules of the unfolded CFTR deletion mutant. This is a multidisciplinary study that nicely combines complementary disciplines to address an important biomedical question, namely the discovery and validation of novel therapeutic molecules for the treatment of the cystic fibrosis. The study is based in an original hypothesis directed to target the interaction of the deletion mutant with proteins such as keratin 8 that appear to prevent its expression in the plasma membrane. Overall, this is a remarkable work, with well-designed experiments, and with conclusions that are supported by the data. A clear strength of the study is the blending of complementary approaches for the characterization of the active compounds that emerged from the in silico screening. Accordingly, I

believe that this manuscript merits publication as an article in EMBO Molec. Med. Nonetheless, there are some concerns to improve the quality and clarity that require attention by the authors before publication.

1. Fig. 2d provides the normalized dose-response curves for the compounds but there is no explanation of how the activity was normalized and whether the maximum activity was different also for the different compounds. Fig 2b and Fig. 2d report that 1uM compound 73100, with an EC50 of 0.8 uM, displays a significantly lower response than compound 407882 that has a 10-fold higher EC50. Thus, the maximum activity appears also an important parameter for comparison between the different compounds to further appreciate their activity.

2. Fig 2c. Have the authors also corrected for the total protein loaded? Regarding to this, on page 10, authors say that compounds do not modify total protein expression. Have authors quantified any housekeeping gene such as actin or tubulin to conclude this? This is not mention anywhere in the manuscript.

3. Fig 3. The synergistic effect of using simultaneously compounds targeting pockets 1 and 2 is not clear. For instance, it can be observed some synergism when using 1 uM of 118208 and 73100 (Fig. 3b and d). However, this synergy is not seen for the combination of 118208 and 407882 that display a rather additive effect. Which would be a plausible explanation for this apparent contradictory result of compounds that bind to the same site? Could it be related to the different activity displayed by compounds 73100 and 407882 at 1 uM (80% vs. 20% of the maximal activation, respectively (Fig. 2D)). It should be noted that a synergistic effect between compounds acting at different sites is better studied by analysis of EC50 displacements rather than using a fix active concentration. A usual synergy study is to use a low active concentration of one compound and then perform a dose response curve of the other compound. Then, EC50 with and without the compound can be compared.

4. Fig 4. Currents traces should be displayed. In addition, it is not convincing why reference compounds (Corr 4a and VX-809) have been used at 10 uM instead of 1 uM for comparison as they were used in iodide fluxes (Fig 2a). Under these conditions, it is very difficult to reach conclusive results on how the activity of the discovered compounds compares with the references. Thus, these measurements should be carried out at the same concentration.

5. In Fig 4a, it is also intriguing that compound 73100 displays a significantly larger activity than 407882, when by using iodide fluxes it was observed that 407822 exhibited stronger response than 73100. Which is the explanation for this contradictory result?

6. In Fig. 4b, the IV curves clearly show the activating effect of the compounds. Intriguingly, it can be also observed that these products alter the reversal potential of the anionic currents. This is quite surprising since it is not expected that this sort of molecules impact the permeability properties of the CFTR channel. Furthermore, the effect on the reversal potential is different for a product that acts on pocket 1 (rightward shift) than for a compound binding to pocket 2 (leftward shift). And the presence of both compounds produces a change that is the average. Do the authors have an explanation for this effect? Did they check the impact of these compounds in wild type CFTR permeability properties?

7. Regarding the mechanism, the authors propose that compound alters the CFTR-K8 interaction and, as a consequence, the channel can be trafficked to the membrane. Although Figure 8 shows an effect on the interaction determined by PLA, these results could be also consistent with alteration of a complex contributed to both CFTR and Keratin 8, but not necessarily a direct effect on their interaction. Thus, a more direct measurement of the impact of compound on CFTR-K8 complex is needed. For instance, the authors could readily use SRP analysis (Colas et al. 2012).

8. The absence of effect of compounds in some cell lines is quite surprising and intriguing. Do the authors have an explanation for this cell-specific effect? The reference provided does not clearly clarify this observation.

© EMBO

Other points to be considered to increase the quality of the study are:

1. Is the VS methodology used in this work different from Kalid et al., 2010? Have authors used a different approaches/protocol, or made some improvements? This fact is not indicated in the introduction or the discussion.

2. Page 4, last sentence: Authors mention modification of keratin 18 network as a potential mechanism; however, this is not well introduced for a non-expert in the field. Why modification of keratin 18 network should contribute to an increase of F508-CTRF in the membrane or increase its activity? This question should be answered.

3. Could authors explain better why the incubation at 27∫C is used as corrector in iodide efflux measurements in HeLa? Include literature, if possible, and mention in the text which is the temperature used to assess the compounds.

4. Authors should describe first all the results with iodide efflux assays, including EC50 results which will give a more complete overview of the pharmacological activity of the compounds. Then, continue with the immunoblot results. So first describe results from figure 2d and then figure 2c. Please, include when possible, 95% limits for the EC50 at least in the text. On page 10, do not compare fold changes at 10uM concentration; compare the EC50 of the compounds. EC50 from reference compound should be included to compare with the active compounds also in the discussion.

5. The description of Figure 4a in pages 11-12 is very confusing when using the fold change since two vehicles are used. Thus, it is suggested that this description be based on the current density values or at least that these values be explicit along with the fold change.

6. Authors assume that detection of fully glycosylated protein band suggest a correct delivery of the channel to the plasma membrane. Do authors have any literature supporting this? If yes, then include it. In addition, it would be more elegant to detect the membrane levels of the protein, where channels are active. Biotinylation or immunocytochemimistry would demonstrate it.

7. Figure 2C should be improved: bands from WT and F508CFTR should be better defined in the figure, it should be clear that compounds are treated only on F508CFTR cells. Band B and C could be named with a proper name to what it define. Statistical analysis applied should be described in the corresponding figure legend.

8. Page 10, last paragraph. Authors evaluate if compounds exhibit also potentiator activity on WT-CFTR cells. Based on supporting figure legend 2, compounds are incubated 24h, and then iodide efflux is induced by incubation with Fsk. It is not clear if compounds are added also with Fsk. However, in the results section, it seems that compounds are only added with Fsk, and they are not pre-incubated for 24h, but from the figure legend it seems that they are added twice 24h before and with Fsk. On the other hand, Gsk is only added with Fsk without pre-incubation. Could authors explain this better?

9. Patch-clamp experiments, page 11 and figure 4. From the figure legend and the results section it is understood that all compounds were tested at 27∫C. Were also experiments with I- efflux measurement performed by incubation of the compounds at 27∫C? If yes, this should be better explain in the manuscript.

10. Patch-clamp experiments, page 11 and figure 4. Authors indicate that DMSO increase 10 folds the current intensity. Did it reach statistical significance? Because in the graph this is not evidence as it does not have any * or ** above the column.

11. Why authors use combination of Fsk/Gsk for iodide efflux experiments and IBMX/Fsk in patch clamp assays?

12. Results on human epithelial cells. Why authors have incubated in CF-KM4 compounds 2h instead of 24h as in HeLa experiments? And why in CF-HBE cells compounds are incubated again 24h? Which is the added value to test the compounds in these two different CF human cells? This could be explained better on the results section and even in the discussion. Figure 5, results with at least a reference compound should have been provided.

13. HDex-MS results, page 15. Why authors named first supplementary figure 7 than supplementary figure 3. In second paragraph: what is control for authors, with vehicle or without nay treatment? What is experimental vs control? It would be better to say, in the presence of the compound versus vehicle or without compound.

14. Reagent and antibodies, cell culture and transeptihelial Cl- current measurement is exactly the same in material and methods section and in supplementary information of material and methods.

15. Authors have included supplementary figure 3 which is related to deuterium uptake experiments, but the manuscript it is not named. Supporting information fig 3 legend: Remove 3A and 3B from the title and replace by 3 A-D.

16. Format from all figures must be homogeneous. Legend for the same treatments should be identical, i.e. in some cases if written NT in other non-treated.

17. All figures in the text are defined with capital letters (i.e Fig 2A) but in the figures they are not in capital letters. Please include which statistical analysis has been performed in all the corresponding figure legends. Indicate in all graphs if data represent mean {plus minus}SD or mean {plus minus}SEM, or write it in material and methods section together with the statistical analysis performed.

18. Figure 4a: remove concentrations from the graph and define them in the figure legend. What means ringer, non-treated? Please, make all figures consistent with the legend for the treatments. In the last bar from the graph, write complete number of the compounds. Please, define in the figure legend as in the text the definition of the CFTR-related current density

19. Figure 6b, same format and column order as the other graphs with bars from the manuscript. Remove N=4 above the columns, since it is in the figure legend. Author could mention in the results section, why amiloride is used at the beginning of the experiment as mentioned in figure 7 legend. Figure 6 legend should be improved, i.e. IBMX and amiloride concentrations are not mentioned.

20. Figure 7: include a graph title for figure 7a and b that help the reader to identify the difference between both graphs. In figure legend, write the complete name for NPD. Effect of a reference compound would have been appreciated.

21. Results, page 9. Selected compounds were incubated for 24h and the CFTR-dependent response was induced by co-treatment with Fsk and Gst, as it is explained in figure legend. It would be appreciated to find this information also in the text. In figure 2A graphs, compound+inh-172 should be identified in the figure legend, not only +inh-172.

22. Sometimes is written Cor4a others Corr4a in the manuscript and graphs, please write it always with the same abbreviation.

23. Figure legend 3: define which ANOVA test

Referee #3 (Comments on Novelty/Model System):

Nice work. I do not have many criticisms.

Referee #3 (General Remarks):

This is a nice manuscript and very comprehensive. I have one suggestion to add some experiments on wt-CFTR for comparison.

1st Revision - authors' response 02 July 2013

Referee #1 (General Remarks):

This work represent a great effort to face a problem beginning with a theoretical approach, followed by a series of biochemical, and functional experiments in cell, tissue a whole-animal models, concluding with a molecular experiments that provides an explanation that are perfectly coherent with the starting molecular models. The main aim of the work, searching for substances to correct CFTR defects, is well accomplished. The whole procedure could be used for searching new, "drugable", compounds for the cystic fibrosis treatment.
The manuscript describes a long series of sophisticated experiments in a perfectly logic sequence. A nonspecialist may have difficulties to follow all experiments, but the conclusion are well explained to put in evidence the nature and aim of each experiment.

We thank the referee for his (her) positive review.

Referee #2 (Comments on Novelty/Model System):

This is a multidisciplinary study that used complementary approaches and technologies to discover and characterize the activity of deltaF508-CFTR correctors with the aim of identifying novel therapeutics for the treatment of cystic fibrosis. In this regard, technical quality, novelty and medical impact are high. The model system used is also appropriate and no ethical concerns are raised.

Referee #2 (General Remarks):

The manuscript by Odolczyk et al. describes the discovery of novel corrector molecules of the unfolded CFTR deletion mutant. This is a multidisciplinary study that nicely combines complementary disciplines to address an important biomedical question, namely the discovery and validation of novel therapeutic molecules for the treatment of the cystic fibrosis. The study is based in an original hypothesis directed to target the interaction of the deletion mutant with proteins such as keratin 8 that appear to prevent its expression in the plasma membrane. Overall, this is a remarkable work, with well-designed experiments, and with conclusions that are supported by the data. A clear strength of the study is the blending of complementary approaches for the characterization of the active compounds that emerged from the in silico screening. Accordingly, I believe that this manuscript merits publication as an article in EMBO Molec. Med. Nonetheless, there are some concerns to improve the

quality and clarity that require attention by the authors before publication.

1. Fig. 2d provides the normalized dose-response curves for the compounds but there is no explanation of how the activity was normalized and whether the maximum activity was different also for the different compounds. Fig 2b and Fig. 2d report that 1uM compound 73100, with an EC50 of 0.8 uM, displays a significantly lower response than compound 407882 that has a 10-fold higher EC50. Thus, the maximum activity appears also an important parameter for comparison between the different compounds to further appreciate their activity.

We agree with the reviewer that both maximum activity and EC_{50} describe the corrector effects. In order to be able to compare the maximal activities of different compounds all dose-response experiments have to be done on the same day and on the same cell batches. This means that 4 different compounds have to be tested at 6-9 concentrations, and repeated 4 times. Our experimental set-up does not allow to do it. Therefore we performed dose-response experiments for different compounds on different days, and chose to normalize the values to the maximal activity taken as 100% (p.10 first paragraph). In the new version we have removed the dose-response for 118208 as we could not normalize it correctly (Fig 2C).

2. Fig 2C (new Fig 2D). Have the authors also corrected for the total protein loaded? Regarding to this, on page 10, authors say that compounds do not modify total protein expression. Have authors quantified any housekeeping gene such as actin or tubulin to conclude this? This is not mention anywhere in the manuscript.

As we agree that our sentence referring to total protein concentration measured before immunoblot analysis (and not by verifying the level of expression of a housekeeping gene) is confusing, we have removed it from the results section. This information is now in supplemental material p7. Regarding relative quantification of mature vs. immature forms of CFTR (band B vs. band C), two methods are frequently used: either the intensity ratio between mature band C and immature band B (e.g. He et al FASEB J 27: 536; 2012), or the ratio between band C and total CFTR (B+C), (van Goor at al PNAS 10: 184843: 2011, e.g. Fig 3; Caputo et al J Pharmacol and Experimental Therapeutics vol 330: 783; 2009 e.g. Fig 1). We have adopted the latter approach by calculating the C/B+C ratio. This is now precised in the supplemental material (Immunoblot section p. 7 and 8).

3. Fig 3. The synergistic effect of using simultaneously compounds targeting pockets 1 and 2 is not clear. For instance, it can be observed some synergism when using 1 uM of 118208 and 73100 (Fig. 3b and d). However, this synergy is not seen for the combination of 118208 and 407882 that display a rather additive effect. Which would be a plausible explanation for this apparent contradictory result of

compounds that bind to the same site? Could it be related to the different activity displayed by compounds 73100 and 407882 at 1 µM (80% vs. 20% of the maximal activation, respectively (Fig. 2D)). It should be noted that a synergistic effect between compounds acting at different sites is better studied by analysis of EC50 displacements rather than using a fix active concentration. A usual synergy study is to use a low active concentration of one compound and then perform a dose response curve of the other compound. Then, EC50 with and without the compound can be compared.

We thank the reviewer for this remark. Accordingly, we have performed a new series of experiments that are presented in the new Fig 3A and B. We have chosen to test 3 concentrations of 73100 and 407882 vs. 1 concentration of 118208 (1 µM). In both cases the synergistic effect was observed at low concentrations (of both 73100 and 407882). For the latter, saturation of the iodide efflux is rapidly reached even at 0.1 µM, while for 73100 it is attained at 1 µM. This is due to the principle of the method, i.e. while the efflux increases, the driving force diminishes preventing its further increase. This also explains why the effect of Fsk+Gst is transient.

4. Fig 4. Currents traces should be displayed. In addition, it is not convincing why reference compounds (Corr 4a and VX-809) have been used at 10 uM instead of 1 uM for comparison as they were used in iodide fluxes (Fig 2a). Under these conditions, it is very difficult to reach conclusive results on how the activity of the discovered compounds compares with the references. Thus, these measurements should be carried out at the same concentration.

As requested by the reviewer, examples of current traces are now displayed (Fig. 4A).

We decided to use the reference compounds Corr-4a and VX-809 at 10µM instead of 1 µM, since in iodide fluxes experiments pretreatment of cells with 1µM Corr-4a was ineffective. Furthermore, treatment of cells with 1µM VX-809 led to a similar level of correction as 10µM treatment. The latter may be explained by the fact that, according to a previous report (van Goor et al PNAS 2011), 1 and 10µM of VX-809 lead to 80-90% of the correction obtained using the optimal concentration (3µM).

5. In Fig 4a, it is also intriguing that compound 73100 displays a significantly larger activity than 407882, when by using iodide fluxes it was observed that 407822 exhibited stronger response than 73100. Which is the explanation for this contradictory result?

We have performed new experiments to increase the number of cells studied, in two groups displaying high SD, i.e. cells treated with compound 73100 and with the VX-809 corrector. The results presented in Fig.4C of the new version show that the efficacy of compound 73100 is still higher but not significantly different from that of compound 407882. We do not have a clear explanation for the

differences in the range of responses observed using either iodide efflux experiments or patch-clamp experiments. It is possible that the greater dispersion of data (S.E.M.) obtained in patch-clamp experiments, probably due to the lower number of cells that can be analyzed, do not allow to unmask statistical differences between groups of cells. On the other hand it is difficult to compare the data obtained from these two approaches since flux does not relate to functional CFTR expression in a linear manner. This is due to driving force issues that lead to transitory shapes of responses. This aspect can partially explain why a synergistic effect could be observed between some compounds using iodide flux experiments and not by measuring currents through open channels. (The same observation can apply for transepithelial current experiments, Fig. 6B, which show neither synergistic nor additive responses to a combination of two compounds).

6. In Fig. 4b, the IV curves clearly show the activating effect of the compounds. Intriguingly, it can be also observed that these products alter the reversal potential of the anionic currents. This is quite surprising since it is not expected that this sort of molecules impact the permeability properties of the CFTR channel. Furthermore, the effect on the reversal potential is different for a product that acts on pocket 1 (rightward shift) than for a compound binding to pocket 2 (leftward shift). And the presence of both compounds produces a change that is the average. Do the authors have an explanation for this effect? Did they check the impact of these compounds in wild type CFTR permeability properties?

We apologize for the confusion generated by the reversal potentials presented initially in Fig.4B. In fact, I/V curves were examples of one cell per group and, unfortunately, they were not representative of the majority of cells tested, in particular for those treated by compound 118208. In the new version, curves have been drawn using mean values of currents recorded at different potentials between -100 and +80 mV. Reversal potential values are in agreement with the reversal potential of a chloride current that can be calculated from the external and internal chloride concentrations used in our experiments (-5.2 mV).

7. Regarding the mechanism, the authors propose that compound alters the CFTR-K8 interaction and, as a consequence, the channel can be trafficked to the membrane. Although Figure 8 shows an effect on the interaction determined by PLA, these results could be also consistent with alteration of a complex contributed to both CFTR and Keratin 8, but not necessarily a direct effect on their interaction. Thus, a more direct measurement of the impact of compound on CFTR-K8 complex is needed. For instance, the authors could readily use SRP analysis (Colas et al. 2012).

We thank the reviewer for this suggestion. We have performed a new series of experiments using the SPR approach. The results show a slight decrease in the binding of K8 to ΔF508-NBD1 and in the association rates when the 407882 or 407882+118208 are present in the K8 pre-incubation buffer as

compared with control conditions. These results are now presented in Fig 8B and Results p. 14, last line and p.15, and mentioned in the discussion on p. 23. The detailed methods for SPR experiments are presented in the supplemental material.

8. The absence of effect of compounds in some cell lines is quite surprising and intriguing. Do the authors have an explanation for this cell-specific effect? The reference provided does not clearly clarify this observation.

We do not have a clear-cut explanation for this effect. One possibility is that the accessibility of compounds to their respective binding sites is different depending on the cell type, which would explain absence of response to both compounds in CF-4KM cells. We agree that this answer is just speculative. As we do not have a precise explanation, and since this series of experiments does not add much to our manuscript, we would agree to remove these results, in case the referee or the editor ask for it.

Other points to be considered to increase the quality of the study are:

1. Is the VS methodology used in this work different from Kalid et al., 2010? Have authors used a different approaches/protocol, or made some improvements? This fact is not indicated in the introduction or the discussion.

Our protocol of virtual screening is significantly different from that presented by Kalid (Kalid et al. 2010). Thus, it is very difficult to compare both strategies in a comprehensive manner in either the introduction or the discussion. The most important conceptual differences were indicated in the primary version of the manuscript. However, according to the Reviewer's question, we decided to highlight those differences in the discussion section. A paragraph has been added accordingly, Discussion p.22, 1st paragraph.

2. Page 4, last sentence: Authors mention modification of keratin 18 network as a potential mechanism; however, this is not well introduced for a non-expert in the field. Why modification of keratin 18 network should contribute to an increase of ΔF508-CTRF in the membrane or increase its activity? This question should be answered.

A couple of sentences clarifying this point have been added to the introduction (pages 4) in the new version.

3. Could authors explain better why the incubation at 27ºC is used as corrector in iodide efflux measurements in HeLa? Include literature, if possible, and mention in the text which is the temperature used to assess the compounds.

We have used 24h incubation at 27°C as a positive control to evaluate the functional rescue by correctors of F508del-CFTR activity since it is accepted as the optimal way to correct F508del-CFTR, based on the original work by Denning et al Nature 1992; 358: 761-764).

On page 9 of the new version a new sentence has been included to clarify this point. Assuming that the maximal correcting effect is obtained after incubation of cells at 27°C, the efficiency of correctors was evaluated by comparing I- fluxes with those obtained at 27°C.

4. Authors should describe first all the results with iodide efflux assays, including EC50 results which will give a more complete overview of the pharmacological activity of the compounds. Then, continue with the immunoblot results. So first describe results from figure 2d and then figure 2c. Please, include when possible, 95% limits for the EC50 at least in the text. On page 10, do not compare fold changes at 10uM concentration; compare the EC50 of the compounds. EC50 from reference compound should be included to compare with the active compounds also in the discussion.

Figure 2 is now presented as suggested by the reviewer. EC50 of VX-809 is added to the text (p10, line 6)

5. The description of Figure 4a in pages 11-12 is very confusing when using the fold change since two vehicles are used. Thus, it is suggested that this description be based on the current density values or at least that these values be explicit along with the fold change.

The description of Fig 4 has been corrected according to the reviewer's remarks, i.e. based primarily on current density values, p.11-12.

6. Authors assume that detection of fully glycosylated protein band suggest a correct delivery of the channel to the plasma membrane. Do authors have any literature supporting this? If yes, then include it. In addition, it would be more elegant to detect the membrane levels of the protein, where channels are active. Biotinylation or immunocytochemimistry would demonstrate it.

It is well accepted that the mature protein is fully glycosylated with a MW of about 170kD (Cheng et al. Cell. 1991;66:1027). We agree with the reviewer that biotinylation or immunocytochemistry would

further support our data. Since the immunoblot analyses show relatively low levels of mature CFTR after treatment, we preferred to use the most sensitive assays, i.e. functional tests, to demonstrate correction by the different compounds. The fact that functional assays constitute the best way to search for ΔF508CFTR correction is wildly accepted by the CF community (see Wang et al Cell 2006 : 127; 803–815, Guggino, W.B., and Stanton, B.A. Nat. Rev. Mol. Cell Biol. 2006 : 7; 426–436).

7. Figure 2C should be improved: bands from WT and ΔF508CFTR should be better defined in the figure, it should be clear that compounds are treated only on ΔF508CFTR cells. Band B and C could be named with a proper name to what it define. Statistical analysis applied should be described in the corresponding figure legend.

Both bands B and C are now defined in the text (p.10 second paragraph), and in the figure legend according to the reviewer's remarks. The statistical test used (unpaired Student's t-test) is indicated in the legend.

8. Page 10, last paragraph. Authors evaluate if compounds exhibit also potentiator activity on WT-CFTR cells. Based on supporting figure legend 2, compounds are incubated 24h, and then iodide efflux is induced by incubation with Fsk. It is not clear if compounds are added also with Fsk. However, in the results section, it seems that compounds are only added with Fsk, and they are not pre-incubated for 24h, but from the figure legend it seems that they are added twice 24h before and with Fsk. On the other hand, Gsk is only added with Fsk without pre-incubation. Could authors explain this better?

We apologize for the mistake in the legend of supporting figure 2. It has been corrected in the new version. To observe potentiator effect we used cells expressing wt-CFTR so no incubation with tested compounds was necessary: CFTR channels are already present in the plasma membrane. Thus, we activated CFTR channels by a cocktail of Fsk + the compound or by Fsk + genistein (genistein is here used as a positive potentiator control).

9. Patch-clamp experiments, page 11 and figure 4. From the figure legend and the results section it is understood that all compounds were tested at 27ºC. Were also experiments with I- efflux measurement performed by incubation of the compounds at 27ºC? If yes, this should be better explain in the manuscript.

Treatment of cells with the different compounds and correctors was performed at 37°C. Treatment at 27°C was used as a positive control, as in iodide flux experiments. This point has been clarified in the Results section (p12) and in the legend of Fig 4.

10. Patch-clamp experiments, page 11 and figure 4. Authors indicate that DMSO increase 10 folds the current intensity. Did it reach statistical significance? Because in the graph this is not evidence as it does not have any * or ** above the column.

The 10-fold DMSO-induced increase in the current density was significant as compared with NT cells and this is indicated next to the corresponding column on Figure 4 in the new version of the manuscript.

11. Why authors use combination of Fsk/Gsk for iodide efflux experiments and IBMX/Fsk in patch clamp assays?

Addition of genistein to Fsk (Fsk stimulates adenylate cyclase and increases cellular cAMP) is required and usually used for iodide flux experiments (for example Schmidt et al, Br. J. Pharmacol., 2008 and references herein). Genistein is a potentiator of CFTR activity that is needed to uncover small responses in ΔF508-CFTR-expressing cells. Since electrophysiological current recording is the most sensitive assay to detect CFTR activity, elevation of cellular cAMP by means of the permeant compound CPT-cAMP or by Forskolin associated with phosphodiesterase inhibitor IBMX is sufficient to detect channel opening. This kind of channel activation is used in most of electrophysiological experiments. In separate patch-clamp experiments we have seen that addition of genistein slightly increases current amplitudes but at the same extent whatever the compound used for cell pre-treatment.

12. Results on human epithelial cells. Why authors have incubated in CF-KM4 compounds 2h instead of 24h as in HeLa experiments? And why in CF-HBE cells compounds are incubated again 24h? Which is the added value to test the compounds in these two different CF human cells? This could be explained better on the results section and even in the discussion. Figure 5, results with at least a reference compound should have been provided.

The effects of F508del-CFTR correctors are not only time- and concentration-dependent, but also cell type-dependent. We carried out preliminary experiments to determine the incubation time leading to the best correction.

For our correctors, we established an optimal treatment of 2h for CF-KM4 and of 24h for Hela cells. This is not the first time that a cell type-dependence of incubation time is described for a F508del-CFTR corrector.

For example, in the study “Anti-inflammatory effect of miglustat in bronchial epithelial cells. Dechecchi et al. (J Cyst Fibros. 2008;7:555-65), the authors demonstrate that miglustat corrects F508del-CFTR after 4h of incubation in IB3 cells and after 24h in CUFI cells.

13. HDex-MS results, page 15. Why authors named first supplementary figure 7 than supplementary figure 3. In second paragraph: what is control for authors, with vehicle or without nay treatment? What is experimental vs control? It would be better to say, in the presence of the compound versus vehicle or without compound.

The figures were re-numbered.

In these experiments, “control” means the corresponding buffer, i.e. an equivalent volume of saline solution for 407882, and DMSO for 118208. This is indicated in the methods in supplementary material. Now, as suggested by the reviewer, it is indicated in the results section as “(NBD1s in the presence of compounds vs. NBD1s without compounds)”, new version p.16.

14. Reagent and antibodies, cell culture and transeptihelial Cl- current measurement is exactly the same in material and methods section and in supplementary information of material and methods.

All methods that are described elsewhere have been removed from the manuscript and are now only in supplementary material.

15. Authors have included supplementary figure 3 which is related to deuterium uptake experiments, but the manuscript it is not named. Supporting information fig 3 legend: Remove 3A and 3B from the title and replace by 3 A-D.

Fig 3 (in the new version Fig 4), is now cited on p16. In the legend of suppl Fig 3A and B (new version suppl 4A and 4B) the changes were done as requested.

16. Format from all figures must be homogeneous. Legend for the same treatments should be identical, i.e. in some cases if written NT in other non-treated.

This has been modified as requested by the reviewer.

17. All figures in the text are defined with capital letters (i.e Fig 2A) but in the figures they are not in capital letters. Please include which statistical analysis has been performed in all the corresponding

figure legends. Indicate in all graphs if data represent mean {plus minus}SD or mean {plus minus}SEM, or write it in material and methods section together with the statistical analysis performed.

Figures are now defined in capital letters. For all the iodide experiments results are presented as mean +/- SEM. This is now indicated in the Material and Methods section in Supplementary Material.

18. Figure 4a: remove concentrations from the graph and define them in the figure legend. What means ringer, non-treated? Please, make all figures consistent with the legend for the treatments. In the last bar from the graph, write complete number of the compounds. Please, define in the figure legend as in the text the definition of the CFTR-related current density

All the improvements suggested by the reviewer for Figure 4 graphs and legend have been performed.

19. Figure 6b, same format and column order as the other graphs with bars from the manuscript. Remove N=4 above the columns, since it is in the figure legend. Author could mention in the results section, why amiloride is used at the beginning of the experiment as mentioned in figure 7 legend. Figure 6 legend should be improved, i.e. IBMX and amiloride concentrations are not mentioned.

The legend has been changed according to the reviewer's suggestion.

20. Figure 7: include a graph title for figure 7a and b that help the reader to identify the difference between both graphs. In figure legend, write the complete name for NPD. Effect of a reference compound would have been appreciated.

The graph titles have been added as suggested. NPD has been fully named in the legend. In our hands neither Corr-4A nor VX-809 were effective.

21. Results, page 9. Selected compounds were incubated for 24h and the CFTR-dependent response was induced by co-treatment with Fsk and Gst, as it is explained in figure legend. It would be appreciated to find this information also in the text. In figure 2A graphs, compound+inh-172 should be identified in the figure legend, not only +inh-172.

These changes have been done as requested.

22. Sometimes is written Cor4a others Corr4a in the manuscript and graphs, please write it always

with the same abbreviation.

We thank the reviewer for careful reading of the manuscript. The same abbreviation has been used in the new version.

23. Figure legend 3:

We used a One-way analysis of variance followed by a Bonferroni post hoc test. This information is now in the figure legends.

Referee #3 (Comments on Novelty/Model System):

Nice work. I do not have many criticisms.

Referee #3 (General Remarks):

This is a nice manuscript and very comprehensive. I have one suggestion to add some experiments on wt-CFTR for comparison.

We have introduced the information about the lack of response of two tested compounds using patch clamp and short-circuit current experiments in the text, p12 and p13.

2nd Editorial Decision 08 July 2013

Thank you for the submission of your revised manuscript to EMBO Molecular Medicine. We have now received the enclosed reports from the Reviewer who was asked to re-assess it. As you will see the s/he is now globally supportive and I am pleased to inform you that we will be able to accept your manuscript pending the following final amendments:

1) Please correct the sentence "dashed line indicate the level of correction...." which appears twice in Figure 3 to "the dashed line indicates the level of correction...."

2) As per our Author Guidelines, the description of all reported data that includes statistical testing must state the name of the statistical test used to generate error bars and P values, the number (n) of independent experiments underlying each data point (not replicate measures of one sample), and the actual P value for each test (not merely 'significant' or 'P < 0.05').

3) Please submit the revised manuscript without the red lettering as this is no longer needed

Please submit your revised manuscript within two weeks. Needless to say, the sooner you do so, the sooner I will be able to accept the next, final version for publication.

***** Reviewer's comments *****

Referee #2 (Comments on Novelty/Model System):

After revising this amended version of the manuscript, I believe that the authors have appropriately replied to all my concerns, carrying out the requested experiments. The quality of the manuscript has significantly increased and it warrants publication in EMBO Medicine. Undoubtedly, this is a novel and original study with relevant results.

Referee #2 (General Remarks):

The authors have addressed all my concerns and the results obtained further support the main conclusions of the study, increasing the quality of the study. I am satisified with the authors reply and believe this study deserves publication in Embo Medicine in its present form.

2nd Revision - authors' response 18 July 2013

On behalf of all the authors, I would like to express our gratitude for your cooperation and kind support during the review process of our manuscript entitled "Discovery of novel ΔF508-CFTR correctors by targeting the specific conformation of nucleotide binding domain" by Odolczyk et al.

Please find enclosed the manuscript files, with all amendments introduced according to your last comments.
