# Supplementary material for: Discovery of novel potent ΔF508-CFTR correctors that target the nucleotide binding domain
Source: EMBO Mol Med. 2013 Aug 27;5(10):1484–501. doi: 10.1002/emmm.201302699 (PMC3799575; doi:10.1002/emmm.201302699)
Supplement: Supplementary file 2 [file emmm0005-1484-SD2.pdf]

# Supporting Information:

## Discovery of novel ΔF508-CFTR correctors by targeting the specific conformation of nucleotide binding domain

Norbert Odolczyk[1], Janine Fritsch[3,4], Caroline Norez[5], Nathalie Servel[3,4], Melanie Faria da Cunha[3,4,], Sara Bitam[3,4,], Anna Kupniewska[3], Ludovic Wiszniewski[6], Julien Colas[3,4], Krzysztof Tarnowski[9], Danielle Tondelier[3,4], Ariel Roldan[8], Emilie L. Saussereau[3,4], Patricia Melin-Heschel[5], Grzegorz Wieczorek[1], Gergely Lukacs[8], Michal Dadlez[9], Grazyna Faure[10], Harald Herrmann[11], Mario Ollero[3,7], Frédéric Becq[5], Piotr Zielenkiewicz[1,2*] and Aleksander Edelman[3,4*]

1 Department of Bioinformatics, Institute of Biochemistry and Biophysics, Polish Academy of Sciences, 02-106 Warszawa, Poland

2 Laboratory of Plant Molecular Biology, Faculty of Biology, Warsaw University, 02-106 Warszawa, Poland

3 INSERM, U845, 75015 Paris, France

4 Université Paris Descartes, Faculté de Médecine, 75015 Paris, France

5 Université de Poitiers, Institut de Physiologie et Biologie Cellulaires, 86 000 Poitiers, France

6 Epithelix SARL, CH-1228 Plan-Les-Ouates, Geneva, Switzerland

7 INSERM, U955, Equipe 21, F-94010 Creteil, France.

8 Department of Physiology, McGill University, Montreal, H3G 1Y6, Canada

9 Laboratory of Mass Spectrometry, Institute of Biochemistry and Biophysics, Polish Academy of Sciences, 02-106 Warszawa, Poland

10 Unité Récepteurs-Canaux; Institut Pasteur, CNRS, URA 2182, 25, rue du Dr. Roux, F-75015, Paris, France

11 Department of Molecular Genetics, German Cancer Research Center, D-69120 Heidelberg, Germany

The authors * should be regarded as joint senior authors

Corresponding authors:

Piotr Zielenkiewicz, Institute of Biochemistry and Biophysics, Polish Academy of Sciences, Pawińskiego 5a, 02-106 Warszawa, Poland, tel. +48225922145, fax. +48226584636, e-mail: piotr@ibb.waw.pl
Aleksander Edelman, INSERM, U845, 75015 Paris, France, tel. +33140615621, fax. +33140615591, e-mail: aleksander.edelman@inserm.fr

# Table of Contents:

**Supporting Information Materials and methods**

**Supporting Information Figures**

*Supporting Information Fig 1.*

*Supporting Information Fig 2.*

*Supporting Information Fig 3.*

*Supporting Information Fig 4A.*

*Supporting Information Fig 4B.*

*Supporting Information Fig 4D.*

*Supporting Information Fig 5A.*

*Supporting Information Fig 5B.*

*Supporting Information Fig 5C.*

*Supporting Information Fig 5D.*

*Supporting Information Fig 6A.*

*Supporting Information Fig 6B.*

*Supporting Information Fig 6C.*

*Supporting Information Fig 6D.*

*Supporting Information Fig 7.*

## Supporting Information Tables

*Supporting Information Table 1.*

*Supporting Information Table 2.*

*Supporting Information Table 3.*

*Supporting Information Table 4.*

*Supporting Information Table 5.*

*Supporting Information Table 6.*

## Supporting Information References

## Supporting Information Materials and methods

### Virtual Screening

#### *Database preparation*

The NCI diversity set I database (http://dtp.nci.nih.gov/branches/dscb/diversity_explanation.html) was retrieved as an sdf file and converted into a mol2 file format using the OpenBabel program (http://openbabel.org). All further preparation was performed using SYBYL 7.3 software (Tripos International, 1699 South Hanley Rd., St. Louis, Missouri, 63144, USA). Atoms and bonds were fixed, hydrogen atoms were added, and Gastiger-Huckle partial charges were assigned. Finally, all structures were minimized using MMFF94s Sybyl's implementation force field [max steps: 500, gradient: 0.05 kcal/(mol*A), minimization method: Powell, with initial simplexing optimization] (Halgren, 1999).

#### *NBD structure preparation for modeling*

Atomic coordinates were retrieved from the molecular dynamic trajectory of ΔF508-NBD1 (Wieczorek & Zielenkiewicz, 2008). The pdb file was created using the trjconv program in the Gromacs package (Hess et al, 2008). AMBER7 FF99 partial charges were loaded using the biopolymer module in Sybyl 7.3, and the structure was saved in the mol2 format. Protein structure was well prepared previously for MD simulation (Wieczorek & Zielenkiewicz, 2008), so that no further optimization was required. Two independent receptors (pockets 1 and 2) were created based on two defined cavities on the ΔF508-NBD1 surface. Pocket 1 was composed of residues G458-A462, K464, C491-S495, S549-A554, S557, D572-L578, E583, V603, S605, S654-E656, R658-S660, L662 and T663, and was enclosed in a cube of 20 x 19 x 14 Å. Pocket 2

was composed of residues M472, R487-I507, G509-S511, R516, D537, I556-A566, and Y569-P574, and was enclosed in a cube of 25 x 20 x 22 Å. To fully prepare both receptors for molecular docking, the molecular surface was calculated according to Richards (Richards, 1977) using the dms program (http://www.cgl.ucsf.edu/Overview/software.html#dms). SPHGEN (Kuntz et al, 1982) was used to create a negative image of the surface, and GRID (Meng et al, 1992) was used to pre-calculate scoring function potential grids (both distributed with Dock 6.1).

### *Virtual Screening procedure*

Two independent protocols were applied for each receptor as follows. The molecular docking program Dock 6.1 (Moustakas et al, 2006) was used to test the conformational space of the ligands inside each cavity (max_orientation: 1000; number of_scored_conformers: 50; all other parameters were set to default). Based on the internal scoring function of Dock, the fifty best conformers per molecule were saved for further minimization into a rigid conformation of the receptors using an MMFF94s force field on the Sybyl dock module [max steps: 2000, gradient: 0.05 kcal/(mol*A), minimization method: Powell, with initial simplexing optimization]. At this point all conformers that have positive electrostatic or steric energy estimations, calculated by a dock module in the Sybyl program (a method similar to the one used in the GRID program; for more details see the Tripos manual) were rejected from further procedure. Next, potential complexes were assessed using different scoring functions: G_Score (Jones et al, 1997), PMF_Score (Muegge & Martin, 1999), D_Score (Kuntz et al, 1982), and ChemScore (Eldridge et al, 1997) in the Sybyl 7.3 CScore module and HMScore, HSScore, and HPScore in the X-score program (Wang et al, 2002). On the basis of results from each scoring function the three best conformations per molecule were saved and subjected to full ligand-receptor minimization [force field: MMFF94s, max steps: 5000, gradient: 0.5 kcal/(mol*A), method: Powell] and then

rescored again, using the selected functions. Furthermore, instead of using consensus scoring methods, we decided to construct a ranking list for each scoring function and treated them independently to select compounds for experimental verification. The top 10 molecules for each scoring function were subjected to a critical visual assessment, and finally twelve molecules (six per cavity) were selected for experimental tests.

**Preparation of figures**

Figures with protein structure visualization were prepared using Visual Molecular Dynamics (VMD) (Humphrey et al, 1996) and The PyMOL Molecular Graphics System, Schrödinger, LLC.

**Reagents and antibodies**

The correcting compounds 118208, 130813 and 73100 were prepared in DMSO (final concentration 0.00002%); compound 407882 was dissolved in water; correctors VX-809 and Corr-4a were prepared in DMSO (final concentration 0.001%). The monoclonal antibodies (mAb) used for CFTR detection were MAB25031 (clone 24-1, R&D systems, USA), MM13-4 (Upstate) and C-terminal CFTR (pCterB, gift from Anil Mehta, University of Dundee, UK). Polyclonal anti-keratin 8 was purchased from Progen.

**Cell culture**

The following cells were used in this study: (i) HeLa cells stably transfected with pTracer plasmid alone (as a control: pTracer), containing WT-CFTR (spTCF-WT) or ΔF508-CFTR (spTCF-ΔF508) provided by Pascale Fanen (Inserm U955, Créteil, France) and grown as described elsewhere (Jungas et al, 2002); (ii) CF-KM4 cell line, obtained by transformation of

primary cultures of CF tracheal gland serous cells homozygous for the ΔF508 mutation with the wt SV40 virus, grown as described elsewhere (Antigny et al, 2008); (iii) human bronchial epithelial cells in primary culture, obtained from ΔF508/ΔF508 patients (CF-HBE) and from non-CF patients after informed consent. Then, they were amplified and seeded at a high density onto microporous filters, and maintained at the air–liquid interface. The cells were cultured at 37°C and 5% $CO_2$ in a humidified atmosphere in a standard tissue culture incubator. The basolateral culture medium (MucilAir culture medium, Epithelix) was replaced every 2–3 days (Crespin et al, 2011). Fully differentiated human air-liquid-interface cultures (MucilAirTM, Epithelix SARL www.epithelix.com) were used for short-circuit experiments.

**Immunoblot experiments**

Cells cultured in 75 $cm^2$ flasks were washed twice with ice cold PBS, scraped in 2 ml of PBS and centrifuged at 600 x g for 5 min. The pellets were suspended in 300 µl of RIPA buffer (50 mM Tris-HCl, 150 mM NaCl, 1% TritonX-100, 1% Na deoxycholate, and 0.1% SDS, pH 7.5) at 4°C for 30 min with agitation. After centrifugation at 15000 x g for 30 min, the supernatants were processed for immunoblot analysis as previously described (Bensalem et al, 2005) with slight modifications. First, protein concentration of different samples was determined using Lowry assay. Samples (40 µg/well) were resolved by 8% SDS-PAGE, transferred onto PVDF membranes, and analyzed by Odyssey infrared imaging system (LI-COR Biosciences, NE, USA) following the manufacturer's recommendations. Membranes were blocked with Odyssey buffer (ScienceTec, Paris, France) for 1 h and hybridized using the monoclonal anti-CFTR Mab24-1 (1/1000). Membranes were then incubated with secondary antibodies (1/10000), and the protein bands were detected (Baudouin-Legros et al, 2012). Relative amount of mature form of CFTR

(band C, ~170kD) to immature form (band B, ~150kD) was evaluated by determining the intensities of band C and B and calculating the ratio C/B+C.

**Iodide efflux experiments**

CFTR chloride channel activity was assayed by measuring iodide ($^{125}I$) efflux from transfected HeLa cells and CF-KM4 cells as described previously (Marivingt-Mounir et al, 2004). Briefly, cells grown for 4 days in 96-well plates were washed twice with 2 ml of modified Earle's salt solution containing 137 mM NaCl, 5.36 mM KCl, 0.4 mM $Na_2HPO_4$, 0.8 mM $MgCl_2$, 1.8 mM $CaCl_2$, 5.5 mM glucose, and 10 mM HEPES, pH 7.4. Cells were then incubated in the same medium containing 1 mM KI and 1 mCi of $Na^{125}I$/ml (NEN Life Science Products) for 30 min at 37°C. After washing, cells were incubated with 1 ml of modified Earle's salt solution for 1 min, following which the medium was removed and saved and quickly replaced with 1 ml of fresh medium. This procedure was repeated every 1 min for 8 min in total. The first three aliquots were used to establish a stable baseline in the efflux buffer. Cells were then co-treated with 10 μM forskolin and 30 μM genistein to increase intracellular cAMP and activate CFTR chloride channels. At the end of incubation, the medium was recovered, and cells were solubilized in 1 N NaOH. Radioactivity was determined using a γ-counter (LKB). The total amount of $^{125}I$ (in cpm) at time 0 was calculated as the sum of cpm for each 1-min sample plus the cpm for the NaOH fraction. The fraction of initial intracellular $^{125}I$ lost during each time point was determined, and time-dependent rates of $^{125}I$ efflux were calculated according to Becq et al. (Becq et al, 1999) using the following equation: $\ln(125It1/125It2)/(t1\ 2\ t2)$, where $^{125}It$ is the intracellular $^{125}I$ at time $t$ and $t1$ and $t2$ are successive time points. Curves were constructed by plotting the rate of $^{125}I$ efflux versus time. Data are presented as the means ± S.E. of 3 separate experiments.

Data were analyzed using the Student's t-test; statistical significance was established if the *p* value was not greater than 0.05. One-way analysis of variance follow by a Bonferroni post hoc test

**Whole cell patch clamp recordings**

The technique for patch clamp recordings in the whole cell configuration has been described elsewhere (Hinzpeter et al, 2006; Tanguy et al, 2008). Stably transfected cells were plated in 35-mm plates that were mounted on the stage of an inverted microscope. Patch-clamp experiments were performed at room temperature with an Axopatch 200A amplifier controlled by a computer *via* a digitdata 1440 interface (Axon Instruments, USA). Pipettes were pulled from hard glass (Kimax 51) using a Sutter micropipette puller, and the tips were fire-polished. Current recordings were performed using the nystatin-perforated patch-clamp configuration. The nystatin stock solution (50 mg/ml) was prepared daily in DMSO. The stock solution was diluted (1:250) with the internal solution, which was sonicated for 1 min. The internal solution contained the following (in mM): 131 NaCl, 2 $MgCl_2$, and 10 Hepes-$Na^+$, pH 7.3, adjusted with NaOH. The bath solution contained (in mM): 150 NaCl, 1 $CaCl_2$, 1 $MgCl_2$, 35 sucrose and 10 Hepes-$Na^+$, pH 7.3, adjusted with NaOH.

Currents were recorded by the application of regular voltage pulses of 60 mV for 1 s, with a holding potential of 0 mV and an interval of 3 s.

To establish the I-V curves, regular voltage pulses were interrupted by a series of 9 voltage jumps (1-s duration each) toward membrane potentials between −100 and +80 mV. CFTR $Cl^-$ currents

were activated using 200 μM 8-(4-chlorophenylthio)-cAMP sodium salt (CPT-cAMP) and 100 μM 3-isobutyl-1-methylxanthine (IBMX).

When maximal stimulation was reached, cells were bathed with 5 μM of the specific CFTR inhibitor $CFTR_{inh}$-172, which was added to the CPT-cAMP solution. CFTR currents were defined as a difference in current amplitude recorded during maximum stimulation by CPT-cAMP after inhibition with $CFTR_{inh}$-172. Data were analyzed using the Student's t-test; statistical significance was established if the *p* value was not greater than 0.05.

**Nasal Potential Difference (NPD) measurements**

The method for nasal potential measurement was adapted from the technique developed for young children (Sermet-Gaudelus et al, 2010). Mice were anesthetized by an intraperitoneal injection of ketamine (133 mg/kg; IMALGENE 1000, MERIAL, France) and xylazine (13.3 mg/kg; Rompun 2%, BayerPharma, France). Mice were positioned on a board tilted 45°, and a paper pad was placed under the nose to avoid suffocation. A subcutaneous needle was connected to an $Ag^+$/AgCl reference electrode by an agar bridge. A double-lumen polyethylene catheter (0.5 mm diameter) was inserted into one nostril (4 mm depth). One lumen was perfused with Ringer solution (in mM: 140 NaCl, 6 KCl, 10 Hepes, 10 glucose, 1 $MgCl_2$, and 2 $CaCl_2$, pH adjusted to 7.4 with NaOH) at 0.15 mL/h. This apparatus was connected to a measuring $Ag^+$/AgCl electrode. The two $Ag^+$/AgCl electrodes were connected to a high-impedance voltmeter (LOGAN research Ltd, United Kingdom). The second lumen was perfused with the following sequence: (1) Ringer solution; (2) Ringer solution containing amiloride (inhibitor of $Na^+$ conductance, 100 μM); (3) Low Chloride Ringer solution, to unmask $Cl^-$ conductances (in mM: 140 Na gluconate, 6 K gluconate, 10 Hepes, 10 glucose, 1 $MgCl_2$, and 6 Ca-gluconate, pH adjusted to 7.4 with NaOH);

and (4) Low Chloride Ringer solution containing $CFTR_{inh}$-172 (5 µM, Calbiochem, Germany) to evaluate the participation of CFTR. Each solution was perfused for at least 3 min at 30-s intervals. The steady-state transepithelial potential, $V_{TE}$, $\Delta V_{TEAmil}$ (difference between $V_{TE}$ and transepithelial potential recorded after perfusion with the amiloride-containing solution), $\Delta V_{TEamilLowCl}$ (difference between $V_{TE}$ and transepithelial potential recorded after perfusion with Low $Cl^-$ plus amiloride-containing solution) and $\Delta V_{TEamilLowClInh-172}$ (difference between $V_{TE}$ and after addition of the CFTR inhibitor to the previous solution) were recorded during the perfusion intervals.

The experimental protocol for compound test was as follows: on day 1 $V_{TE}$ was measured before administration of compound 407882. Mice were left recovering for a week to repair the epithelial layer possibly damaged by perfusion catheters. On day 7 and 8, the compound 407882 (20µL at 10µM) was administrated intranasally twice within 48 h, the second time 24 h before $V_{TE}$ measurements at 0.1 µmol each/mouse. On day 9, i.e. 48 h after treatment, $V_{TE}$ was measured again.

**Transepithelial $Cl^-$ current measurments**

The ion channel activity of CFTR across an epithelial cell layer in air-liquid conditions was assessed by measuring short circuit current changes ($\Delta I_{sc}$) in a modified Ussing chamber (EM-RSYS-8; Physiologic Instrument) connected to a multichannel current/voltage clamp (VCC8; Physiologic Instrument). Signals were recorded using the dedicated program of Physiologic Instrument (acquisition of data and analysis). For each compound, the Ussing chamber measurement was performed on MucilAir-CF from two CF donors bearing ΔF508/ΔF508 mutation, in duplicate, preincubated during 48 hours with 1 µM of compounds 118208 or 408882

or a combination of both (1 μM each). The final concentration of DMSO was 0.00002 %. The solution at the basal side was 5 ml of 37°C Krebs-bicarbonate-Ringer containing (in mM): 140 $Na^+$, 120 $Cl^-$, 5.2 $K^+$, 1.2 $Ca^{2+}$, 1.2 $Mg^{2+}$, 2.4 $HPO_4^{2-}$, 0.4 $H_2PO_4^-$, 25 $HCO_3^-$, and 5 glucose, circulated with 95% $O_2$-5% $CO_2$ gas, pH 7.4. $\Delta I_{sc}$ were measured using a transepithelial $Cl^-$ gradient. In the apical solution, $Cl^-$ was replaced by gluconate. Ion channel activity and $\Delta I_{sc}$ was monitored and followed using the sequential addition of sodium channel inhibitor Amiloride (100 μM), CFTR activator cocktail IBMX/Fsk (50 μM/25 μM) and the specific CFTR channel inhibitor Inh-172 (20 μM).

**Proximity ligation assay**

Cells were fixed with cold acetone and analyzed using the Duolink™ kit (Eurogentec, Angers, France) according to manufacturer's instructions. Briefly, slides were pre-washed in PBS, and incubated with blocking solution. Samples were then incubated with primary antibodies against C-terminal CFTR (polyclonal anti-CterB, provided by Anil Mehta, University of Dundee, UK) and K8 (Progen), at 1:100 and 1:10 dilutions respectively. Secondary antibodies conjugated with oligonucleotides (PLA probe MINUS and PLA probe PLUS) were added to the reaction tube and incubated. The oligonucleotides contained in the hybridization solution hybridized to the two PLA probes if they were at <40 nm. A ligase (Ligation Solution), nucleotides and polymerase were added sequentially, allowing formation of a rolling-circle amplification product detected by labelled oligonucleotides in case of proximity. The signal was visible as a distinct fluorescent dot and analyzed by fluorescence microscopy (excitation at 557 nm and emission at 563 nm) using 640 oil objectives (Leica TCS SP5 Confocal Microscopy System).

**Surface plasmon resonance**

ΔF508NBD1 was purified according to the protocol described in (Rabeh et al, 2012). Keratin 8 was purified according to Herrmann et al. (Herrmann et al, 2002). Three thousand four hundred RU of ΔF508-NBD1 and 2900 RU of WT-NBD1 were immobilized. Firstly, the interaction between K8 and NBD1 (WT and ΔF508) was monitored by injecting 0.1 mg/ml of K8 in running buffer [50 mM Tris-HCl at pH 7.4, 150 mM NaCl, 5 mM MgCl2, 1 mM $ATP_{Mg}$, 1 mM DTT and 0.005% (w/v) surfactant P20]. Then, for competition experiments, the indicated running buffer was supplemented with 180 μM 407882 and 180 μM 118208 (to saturated immobilized NBD1) and K8 (0.1 mg/ml) was diluted in the same buffer with the two molecules (407882 and 118208) (the molar ratio between K8/small molecules was 1/100).

The initial slope of RU changes ΔRU/s was calculated by measuring the changes in RU between 1 and 11 sec. The maximal change in RU under the different experimental conditions was taken at 50 sec.

## Hydrogen deuterium exchange MS

### *List of peptic peptides*

In order to establish the list of peptic ΔF508-NBD1 peptides (Supporting Information Fig 4) which served for subsequent HDex MS data analysis the following reaction was performed: 5 μl of the ΔF508-NBD1 stock solution (140 μM) was mixed with 45 μl of reaction buffer (50 mM Tris pH 8, 300 mM NaCl). Next, the sample was acidified by adding 10 μl of stop solution (2 M glycine pH 2.5). The peptides were obtained by pepsin digestion using an immobilized pepsin resin 2.1 mm x 30 mm column [Porozyme, ABI, Foster City, CA] in the temperature controlled compartment of the HDX manager set at 20 °C at 200 μL/min flow of 0.07 % formic acid in water as a mobile phase. Digested peptides were passed directly to the C18 trapping column [ACQUITY BEH C18 VanGuard precolumn, 2.1 mm x 5 mm, 1.7 μm resin, , Waters, Milford, MA].Trapped peptides were then subjected to reverse-phase chromatography using UPLC system and C18 column [Acquity UPLC system, BEH, C18 column, 1.0 x 100 mm, 1.7 μm resin, Waters, Milford, MA] using a 6% – 40% gradient of acetonitrile in 0.1% formic acid at 40 μL/min, which was supplied by a nanoACQUITY Binary Solvent Manager. All fluidics, valves and columns were maintained at 0.5 $^{o}$C using the HDX Manager [Waters, Milford, MA]. The column outlet was coupled directly to the ion source of SYNAPT G2 HDMS mass spectrometer [Waters, Milford, MA] working in Ion Mobility mode. Lock mass was activated and carried out using leucine-enkephalin [Sigma]. For protein identification mass spectra were acquired in $MS^E$ mode over the m/z range of 50 – 2000. The spectrometer was calibrated on a weekly basis using standard calibrating solutions [Waters, Milford, MA]. Peptides were identified using ProteinLynx Global Server software [Waters, Milford, MA]. The list of identified peptides containing the

following features: m/z, retention time and ion mobility drift time was passed to the DynamX HD exchange data analysis program [Waters, Milford, MA].

### *Hydrogen-deuterium exchange (HDex) reaction*

4 µl of ΔF508-NBD1 or WT-NBD1 in stock solution was mixed with 1 µl of 3.5 mM 118208 or 7 mM 407882 compounds dissolved in appropriate buffer (7% DMSO or 0.013 M NaOH in reaction buffer). Control reactions were done by adding the equivalent amounts of respective compound buffers. The HD exchange reaction was performed using same reactions schemes as described above for establishment of the peptic but using buffers in which $H_2O$ was replaced by $D_2O$ [99.8%, Armar Chemicals, Switzerland]. The pH of the experimental and control mixtures was precisely controlled. No difference in pH was observed. The exchange reactions and controls were carried out for 10 s, at room temperature.

Two control experiments were carried out to take into account in- and out-exchange deuterium artifacts, as described previously (Kupniewska-Kozak et al, 2010).

### *HDex data analysis*

In the first step of the data analysis the average masses of peptides after HD exchange reaction were calculated automatically using DynamX software, based on the peptic peptide list obtained from PLGS program, and further on filtered in DynamX program with the criteria shown in Supporting Information Table 3.

In the second step of analysis the MS spectra after HDex reaction and control in-exchange and out-exchange experiments were processed by DynamX software using parameters presented in

Supporting Information Table 4. The average masses of peptides after HDex experiment ($m_{ex}$) and after two control experiments ($m_{ex0}$ and $m_{ex100}$) obtained from the automated analysis in DynamX were than verified by visual inspection. Ambiguous isotopic envelopes were discarded from further analysis. Final data were exported to the Excel (Microsoft) calculation spreadsheet for HD exchange mass shifts and fraction of exchange calculation. The fraction of exchange (f) of a given peptide was calculated by taking into account both control mass values, following the formula:

$$\%D(\text{deuterium}) = (m_{ex} – m_{ex}^{0}) / (m_{ex}^{100} – m_{ex}^{0})*100 \qquad \text{(Eq. 1)}$$

Differences between HDex level in ΔF508-NBD1 or WT-NBD1 in the presence and absence of compounds where ploted on histograms (Supporting Information Fig 4). The differences in HDex level were considered as significant if not enclosed in area of the mean value of changes ± standard deviation ( $\bar{x} \pm \sigma$)

## Supporting Information Figures

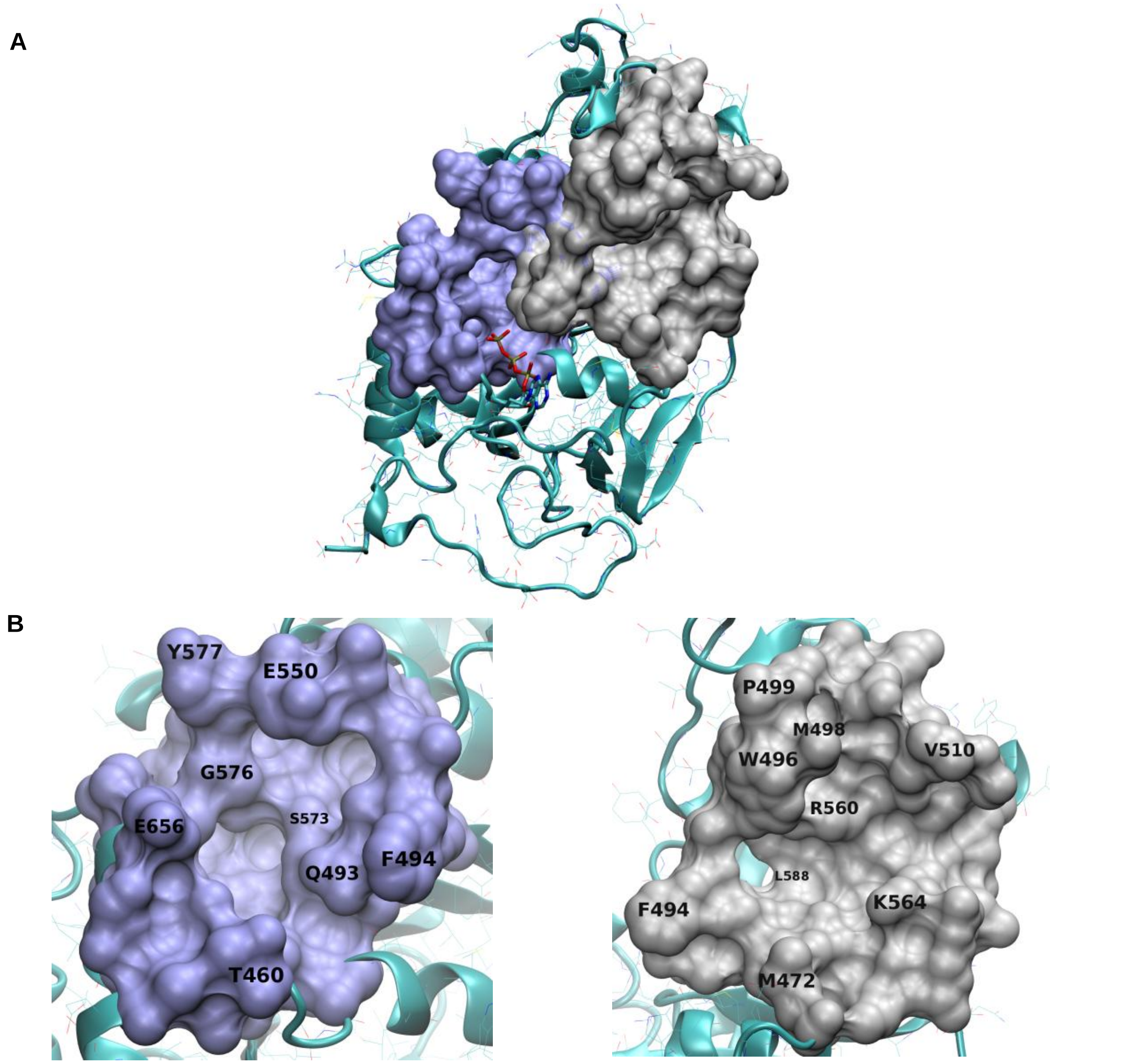


**Supporting Information Fig 1.** Two binding pockets on the ΔF508-NBD1 surface. Two deep cavities identified on the ΔF508-NBD1 surface of unique conformation were treated as two independent receptors for structure-based VS studies. (**A**) Both cavities were indicated in the full structure of NBD1 as solvent exposure surface. ATP bound to NBD1 is also shown. (**B**) The residues within pockets 1 and 2 were labeled and colored ice blue and silver, respectively.

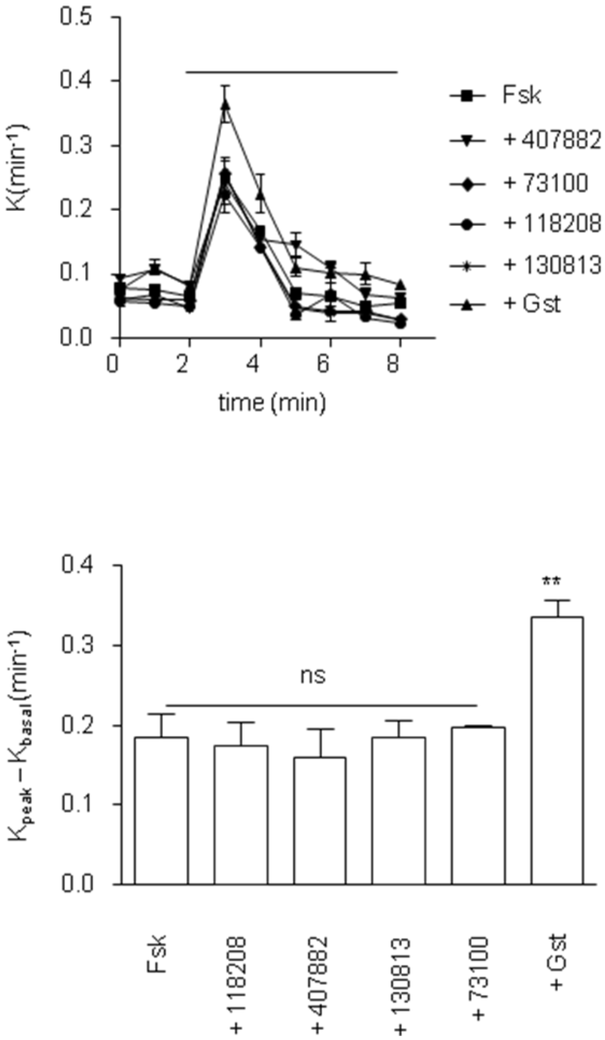


**Supporting Information Fig 2.** (**A**) Representative iodide efflux curves obtained in HeLa cells stably transfected with WT-CFTR. The CFTR-dependent response was induced by treatment with 10 μM forskolin (Fsk) alone or Fsk plus 1 μM of the indicated compounds or Fsk plus 30 μM genistein (Gst), as indicated by the horizontal bar above each trace. (**B**) Histogram showing the peak amplitude of stimulated iodide effluxes in cells treated with the indicated drugs. The values represent the mean + s.e.m. of 3 independent experiments; (**) indicates $P$=0.0006.

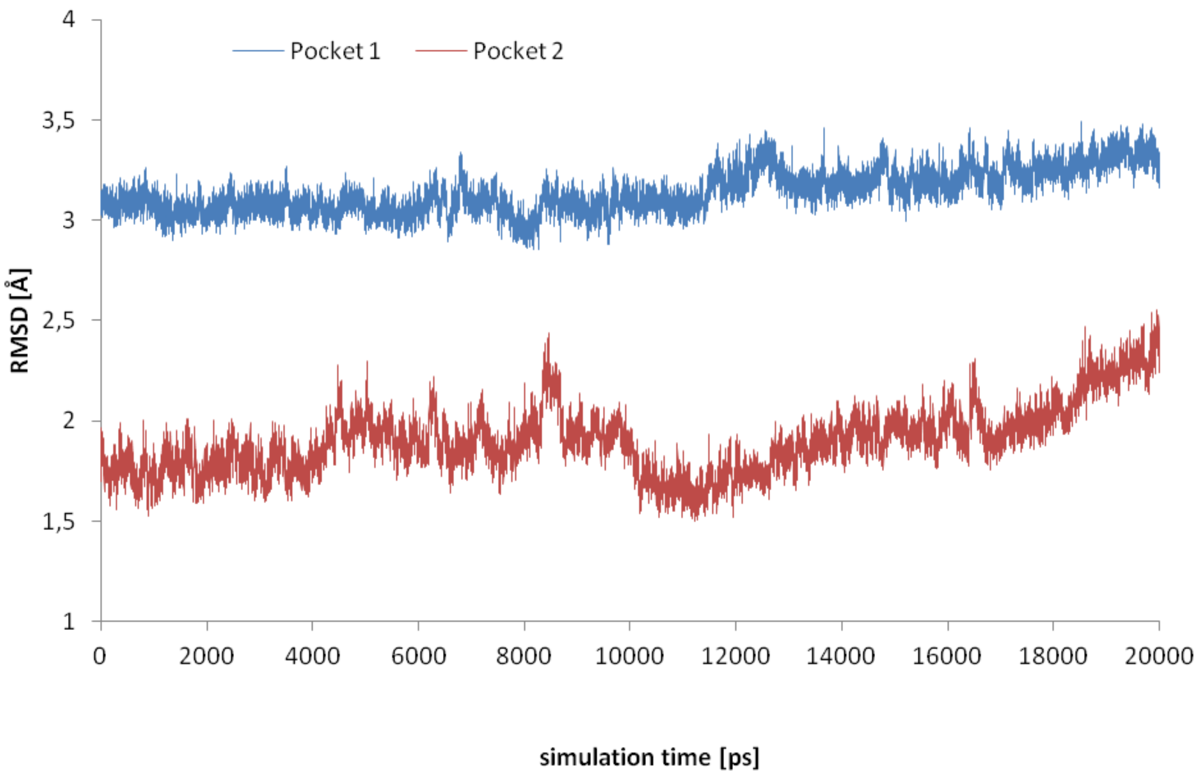


**Supporting Information Fig 3.** The local analysis of WT-NBD1 trajectory only in the areas of two binding regions. Residues of pocket 1 and 2 from docking frame, were fitted independently onto all frames from WT-NBD1 trajectories, and root-mean-square-deviation(RMSD) were calculated. The lowest RMSD value WT *vs* ΔF508 (docking frame) was 2.9 Å and 1.5 Å for pocket 1 and pocket 2 respectively. This suggest that pocket 1 exists only in ΔF508-NBD1, whereas pocket 2 is present in both versions of NBD1. Fitting was performed using the McLachlan algorithm (McLachlan, A.D., 1982 ``Rapid Comparison of Protein Structures", Acta Cryst A38, 871-873) as implemented in the program ProFit (Martin, A.C.R., http://www.bioinf.org.uk/software/profit/).

**Supporting Information Fig 4A.**

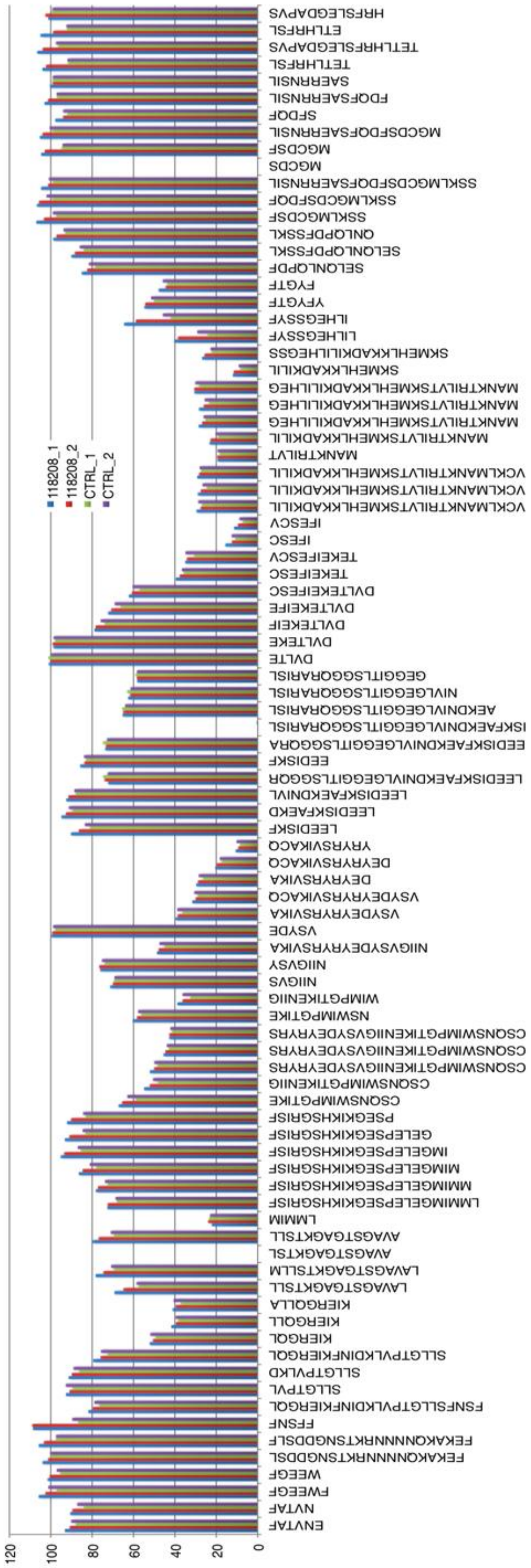

**Supporting Information Fig 4B.**

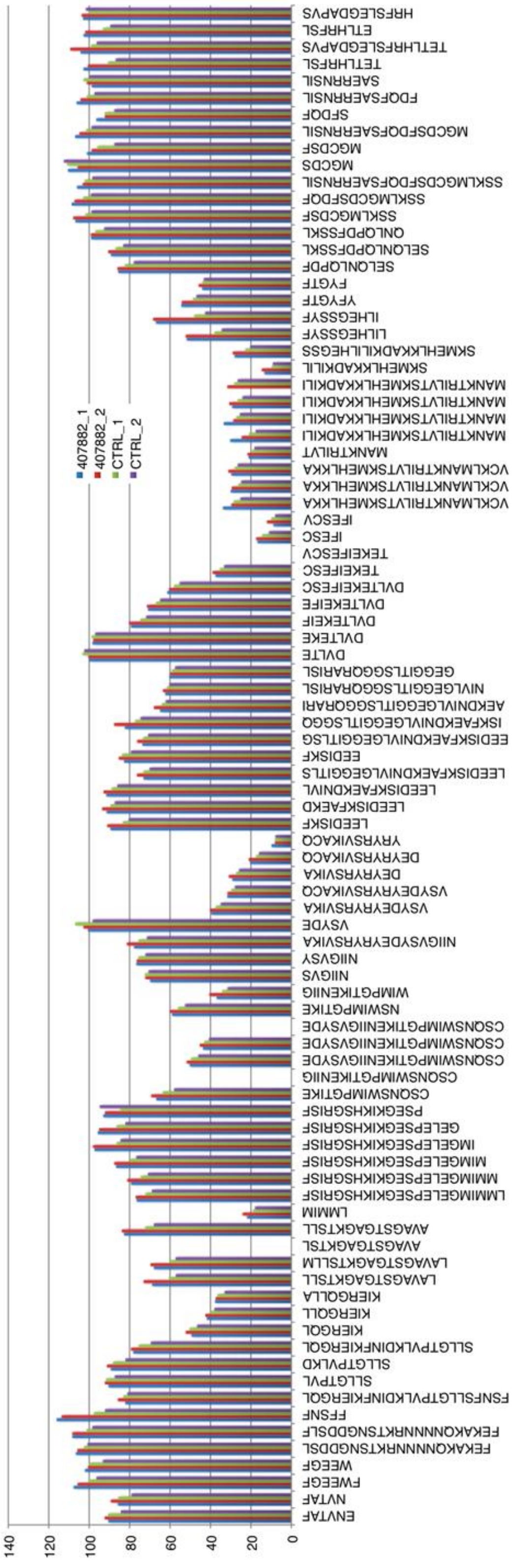

**Supporting Information Fig 4C.**

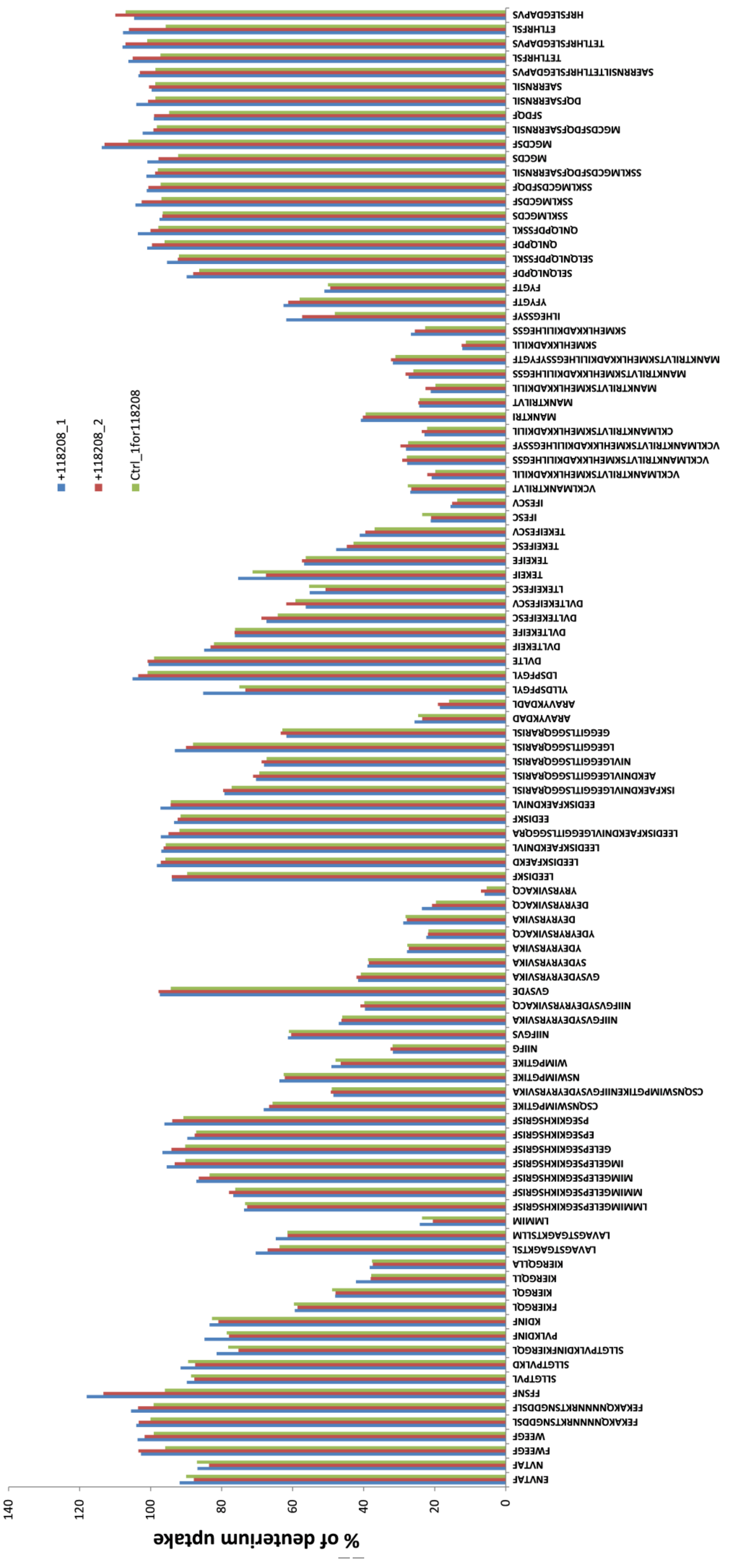

**Supporting Information Fig 4D.**

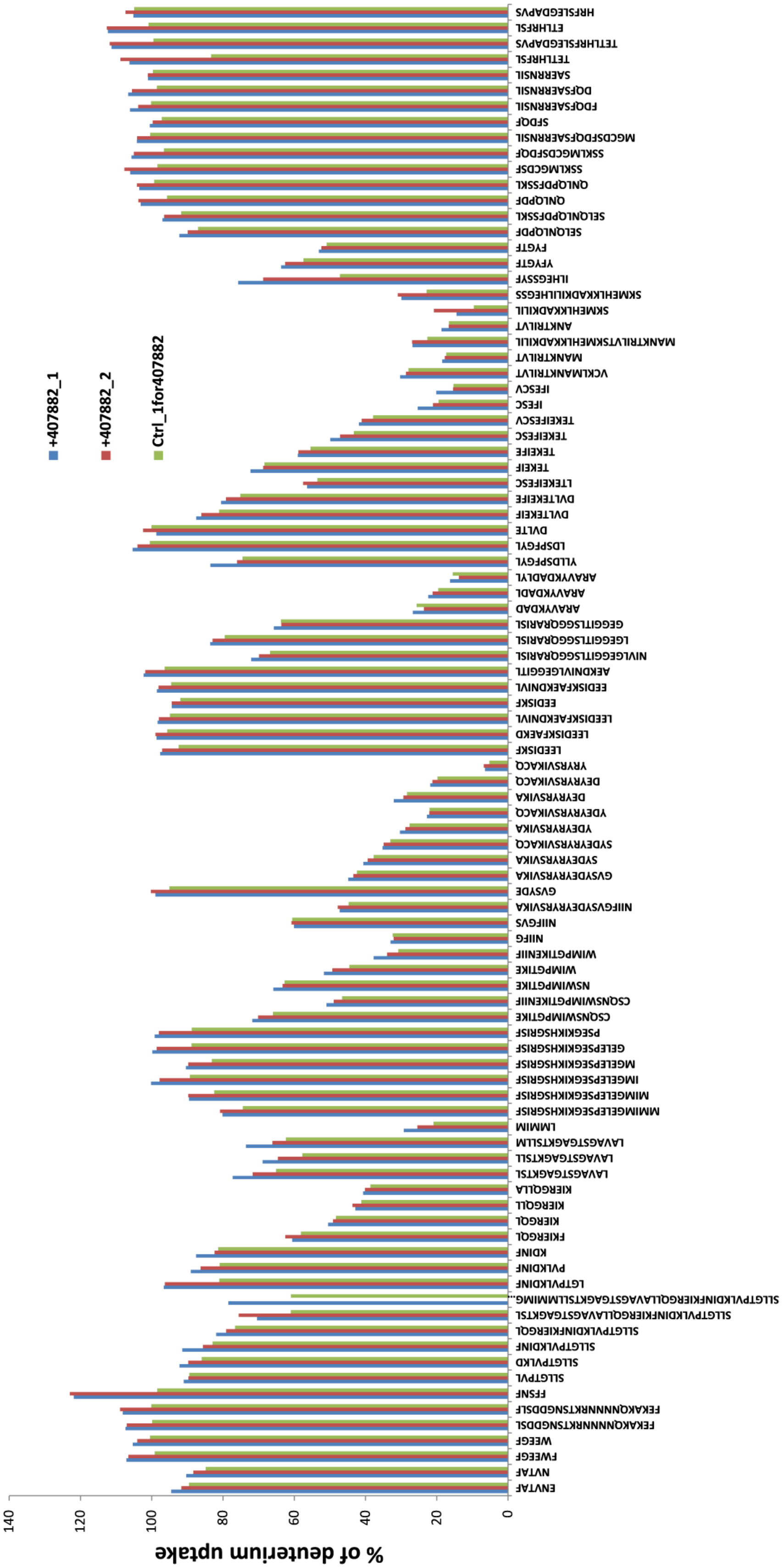

**Supporting Information Fig 4A-D.** Histogram of deuterium uptakes in ΔF508-NBD1 in the presence of compounds 118208 or 407882. The uptakes are schematically represented in a histogram as % of maximal deuterium uptake for each peptic peptide listed in X axis. For each peptide 2 bars (blue and red) correspond to two different HDex-MS experiments measured after 10 s of reaction in the presence of 140 μM 118208 for ΔF508-NBD1 (**A**) and for WT-NBD1 (**C**) whereas 70 μM 407882 was used for ΔF508-NBD1 (**B**) and for WT-NBD1 (**D**). Bars green and violet correspond to controls in the presence of 0.14% DMSO for compound 118208 and 0.26 mM NaOH as indicated in Supporting Information Materials and Methods,.

**Supporting Information Fig 5A.**

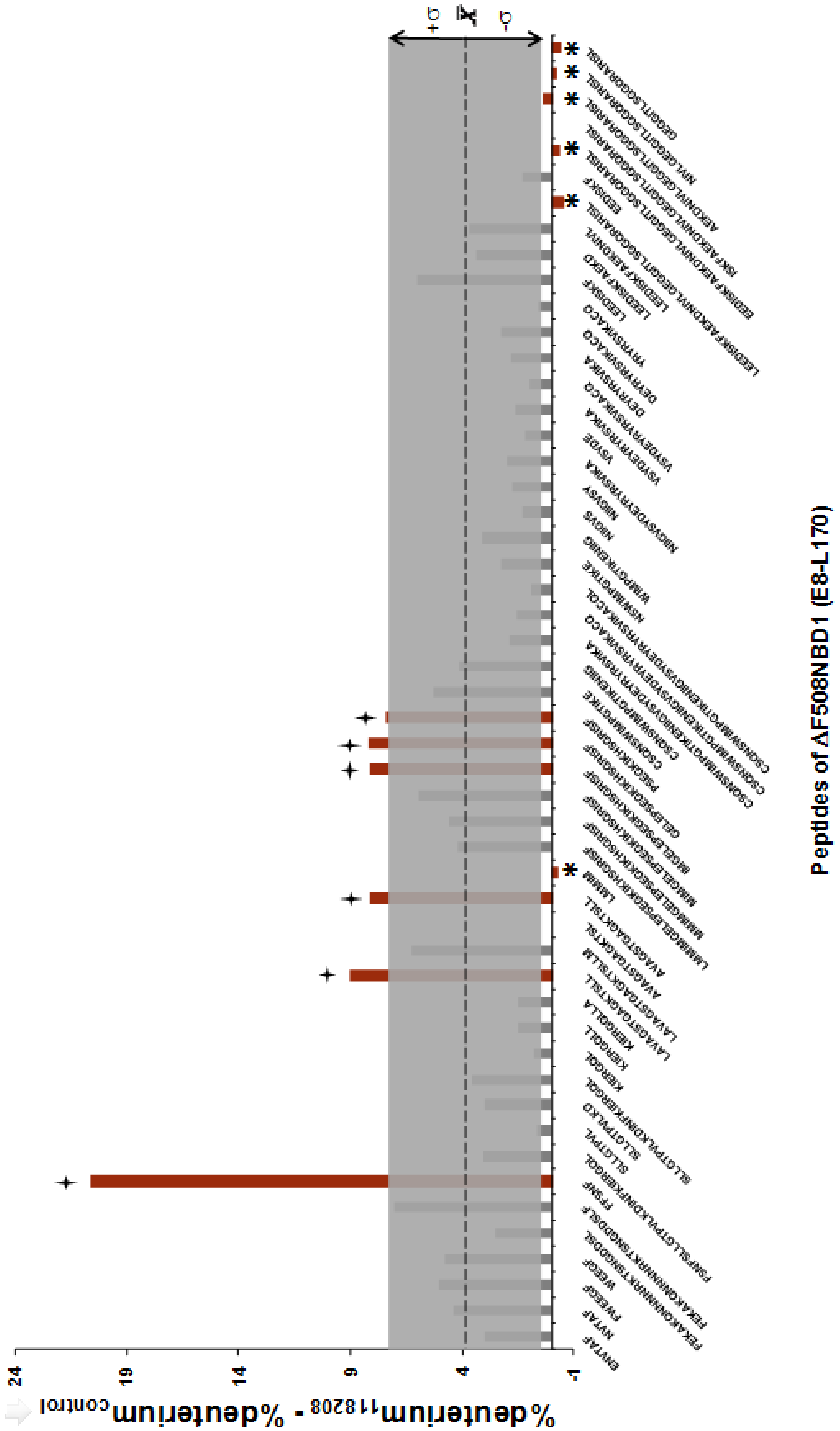

**Supporting Information Fig 5B.**

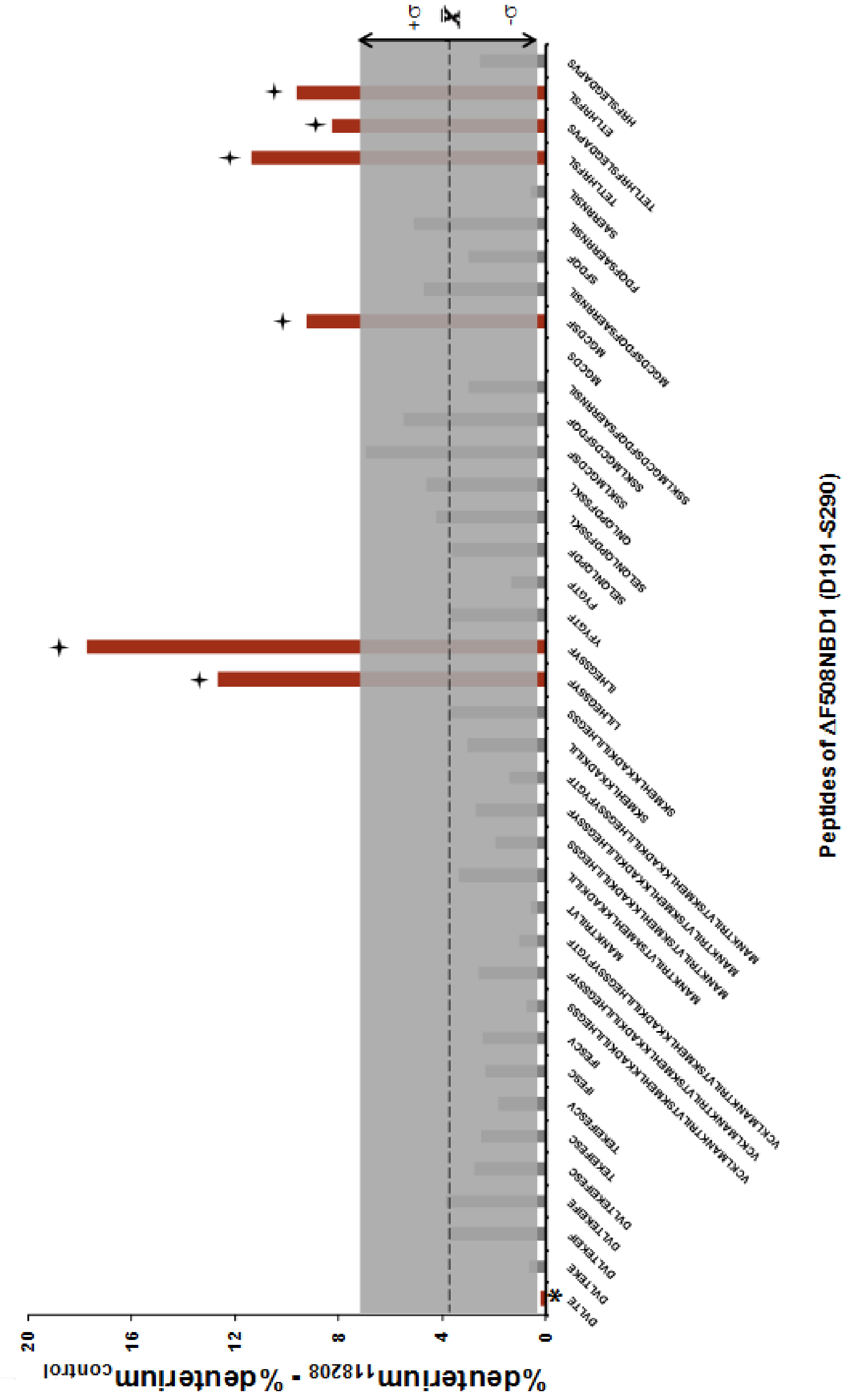

**Supporting Information Fig 5C.**

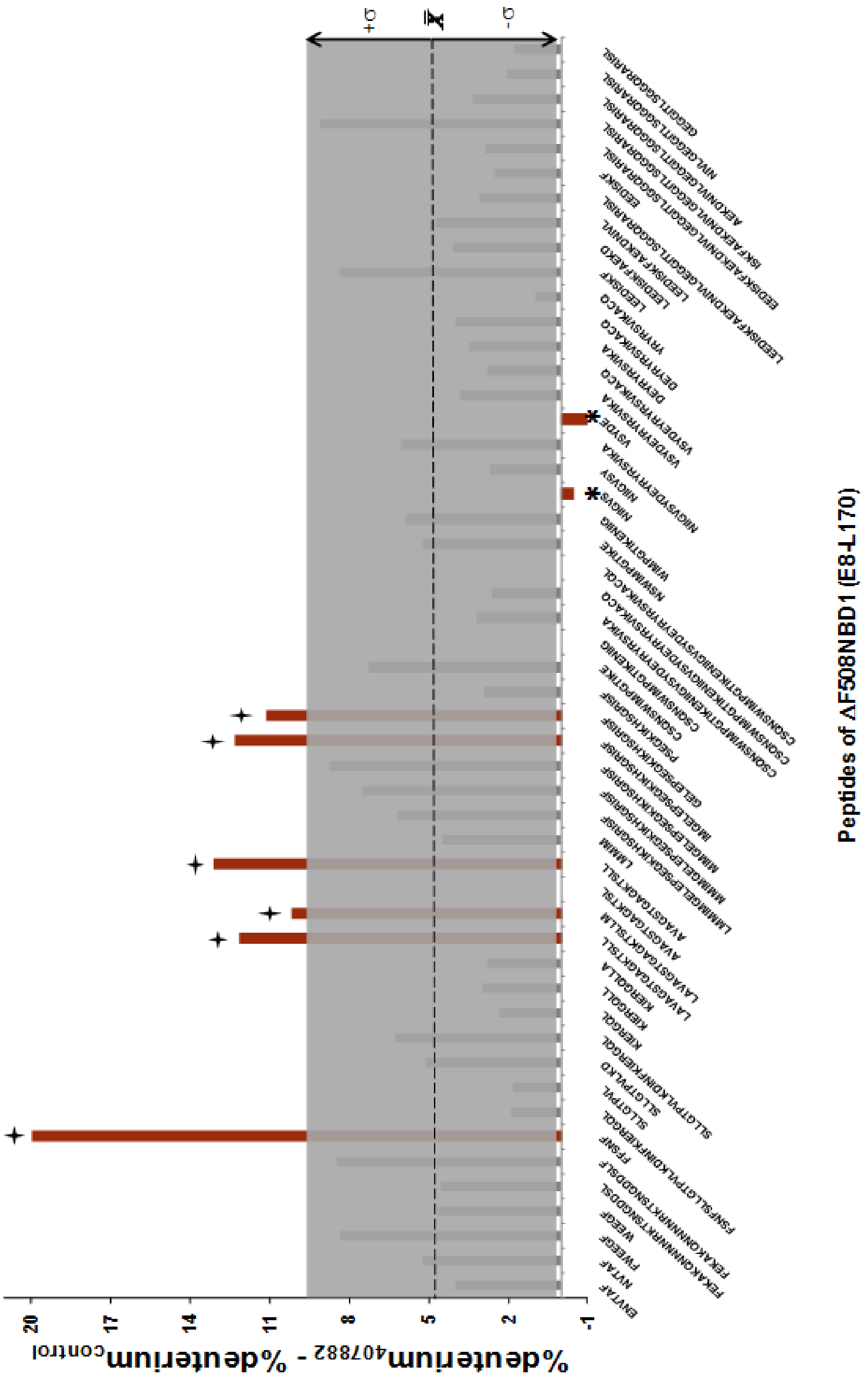

**Supporting Information Fig 5D.**

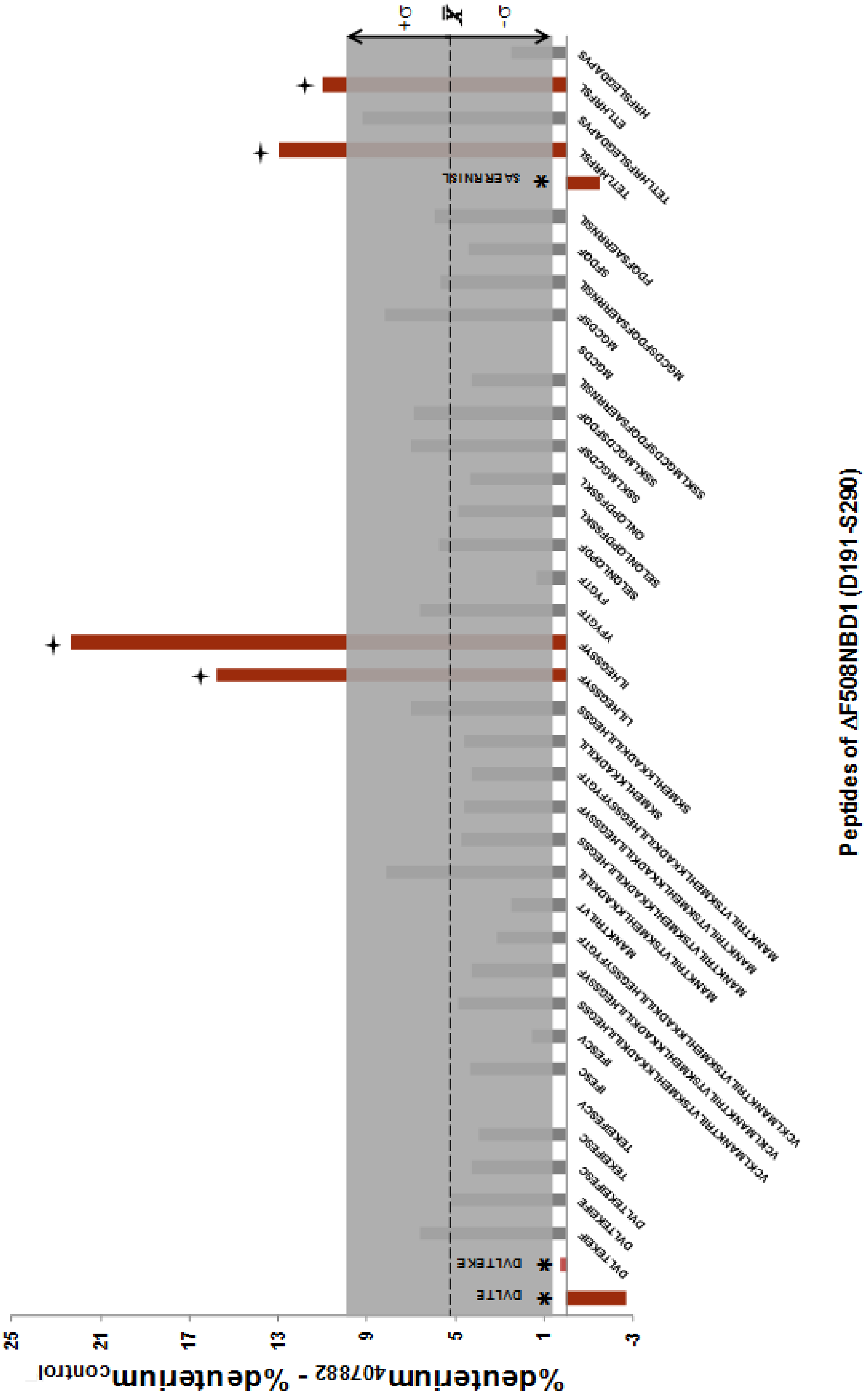

**Supporting Information Fig 5 A-D.** Differences in deuterium uptake between ΔF508-NBD1 incubated with 118208 (**A**)/(**B**) or 4078823 (**C**)/(**D**) compounds $\%D_{drug}$ - $\%D_{control}$ (% deuterium $_{drug}$ -% deuterium $_{control}$ ). Peptides of ΔF508-NBD1 concerning $\%D_{drug}$ - $\%D_{control}$ are presented in two panels for representation purposes. On (**A**) and (**C**) have been shown peptides from E8-L170 of ΔF508-NBD1 whereas on (**B**) and (**D**) peptides from D191-S290 of ΔF508-NBD1. Dashed line represents mean value of $\%D_{drug}$ - $\%D_{control}$ ( $\bar{x}$ ), gray surface represents the region comprised between $\bar{x} \pm$ SD (σ). „*" corresponds to peptides which are outside $\bar{x}$ -σ: „+" indicates peptides which are outside $\bar{x}$ + σ.

**Supporting Information Fig 6A.**

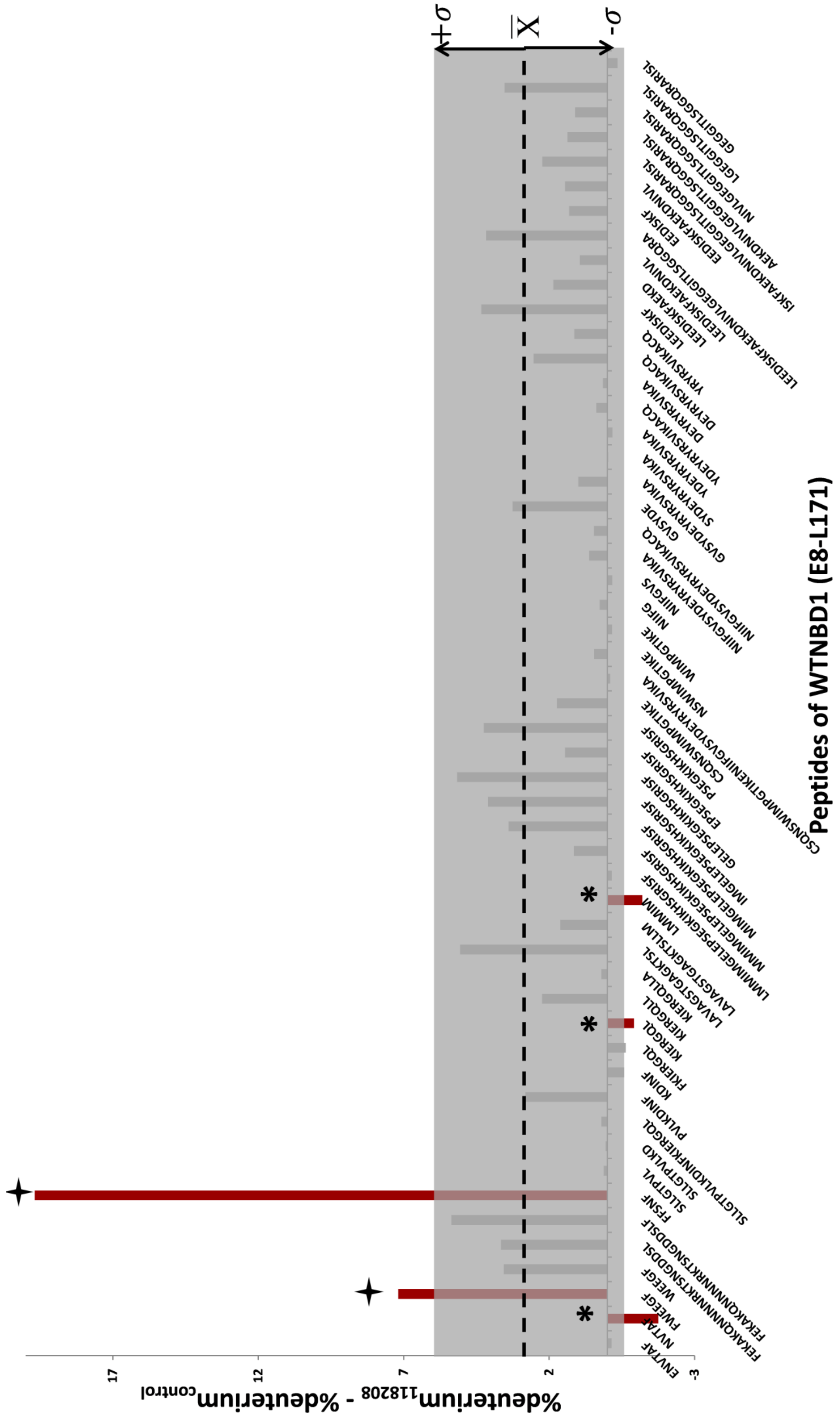

**Supporting Information Fig 6B.**

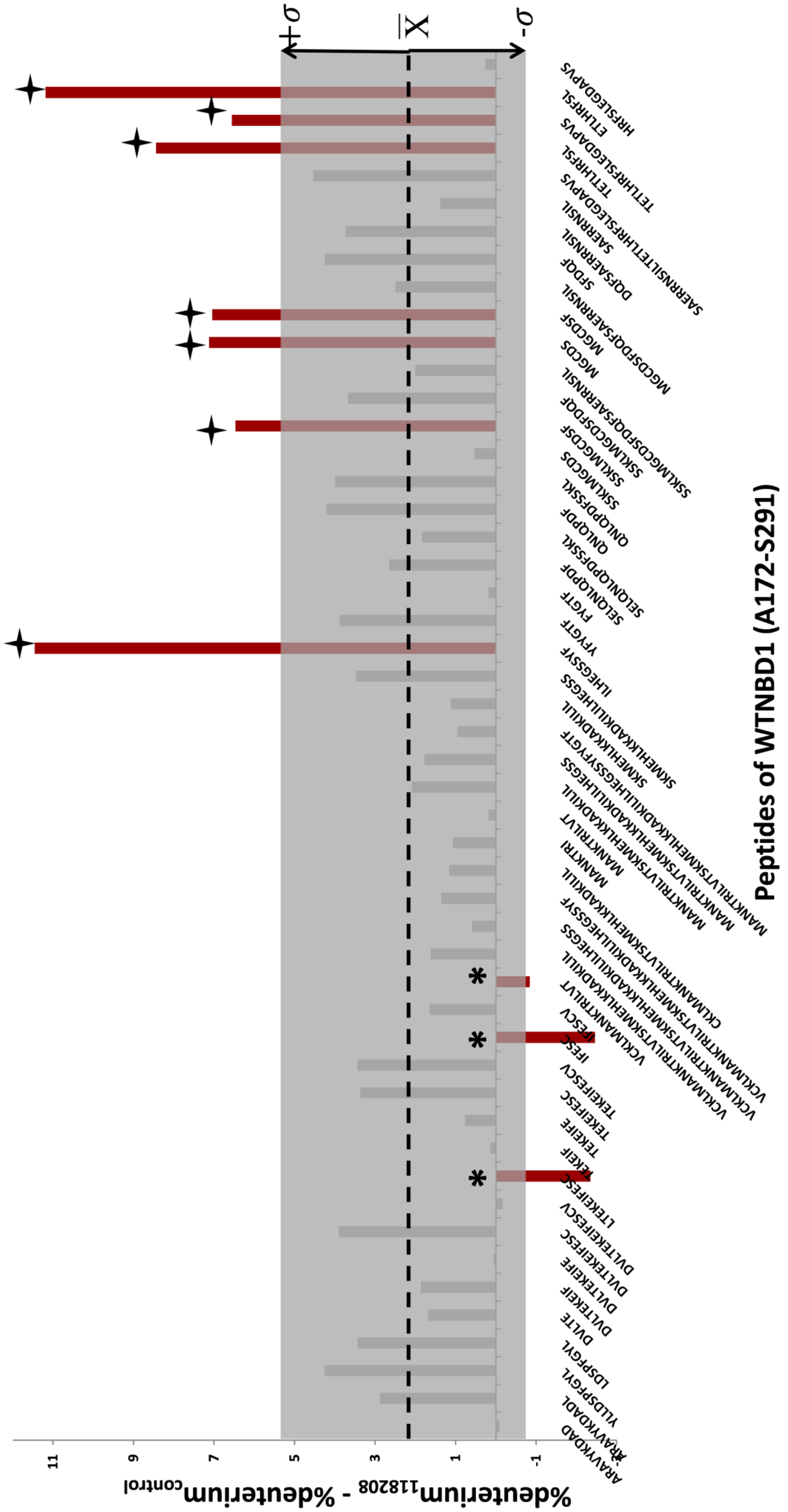

**Supporting Information Fig 6C.**

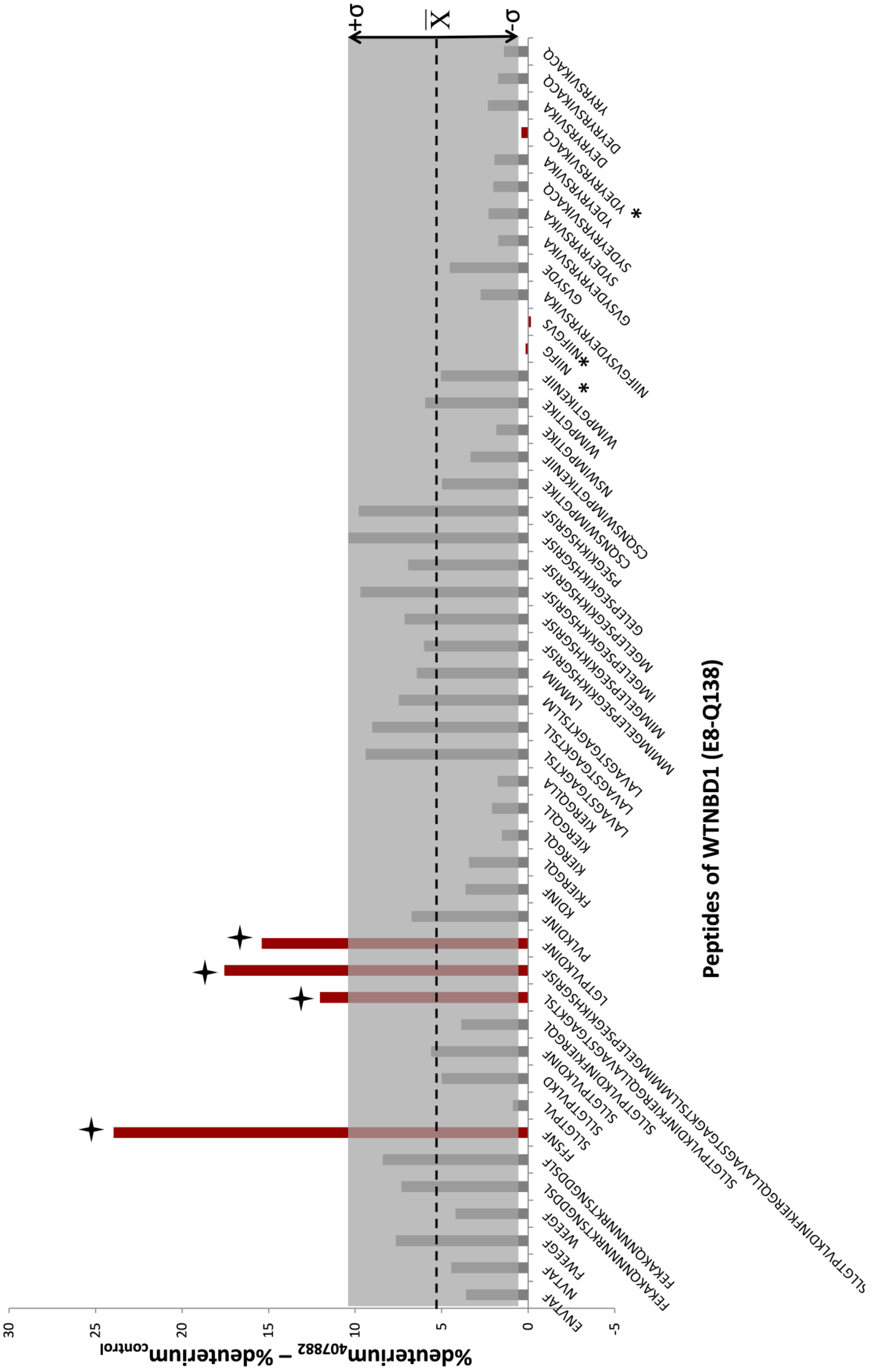

**Supporting Information Fig 6D.**

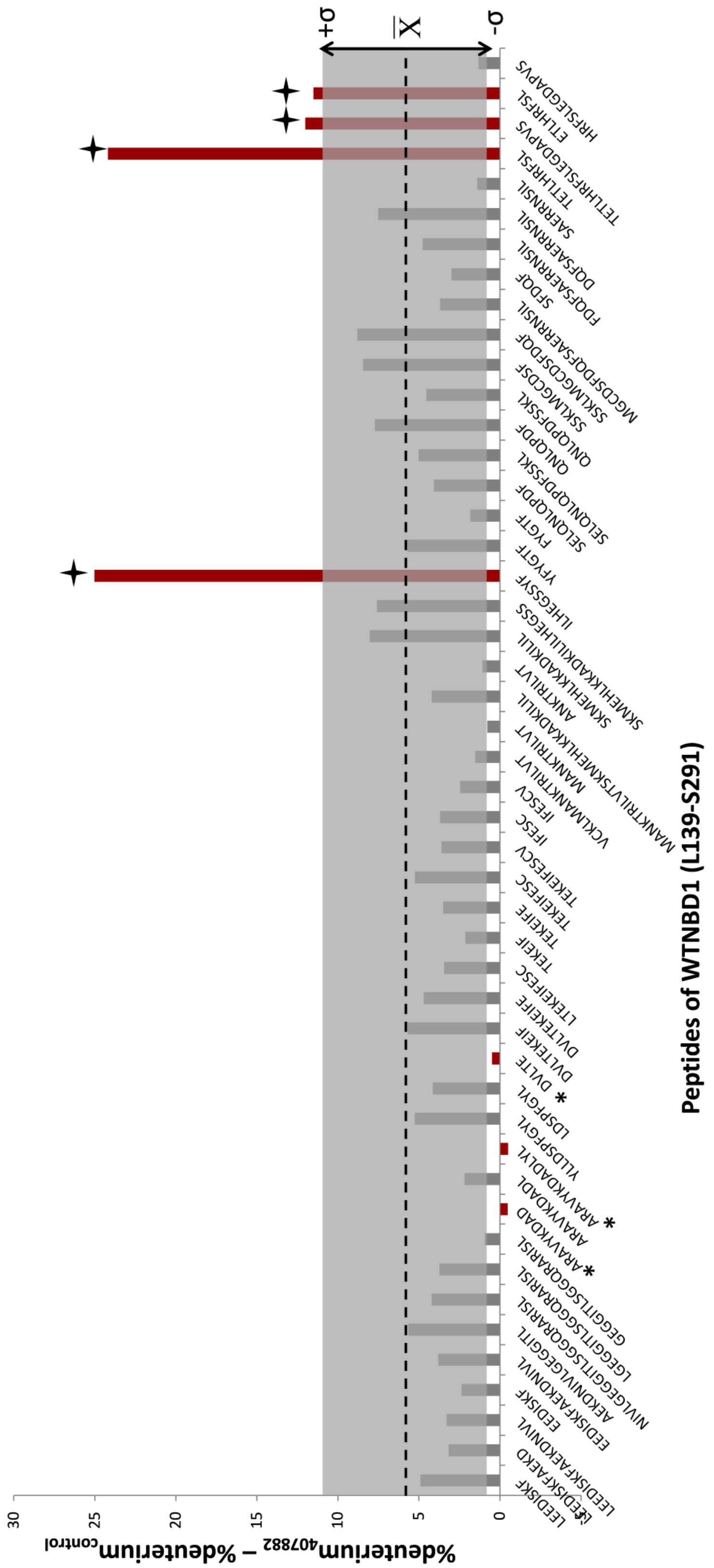

**Supporting Information Fig 6 A-D.** Differences in deuterium uptake between WT-NBD1 incubated with 118208 (**A**)/(**B**) or 4078823 (**C**)/(**D**) compounds $\%D_{drug}$ - $\%D_{control}$ (% deuterium $_{drug}$ -% deuterium $_{control}$ ). Peptides of WT-NBD1 concerning $\%D_{drug}$ - $\%D_{control}$ are presented in two panels for representation purposes. On (**A**) and (**C**) have been shown peptides from E8-L170 of WT-NBD1 whereas on (**B**) and (**D**) peptides from D191-S290 of WT-NBD1. Dashed line represents mean value of $\%D_{drug}$ - $\%D_{control}$ ( $\bar{x}$ ), gray surface represents the region comprised between $\bar{x} \pm$ SD (σ). „*" corresponds to peptides which are outside $\bar{x}$ -σ: „+" indicates peptides which are outside $\bar{x}$ + σ.

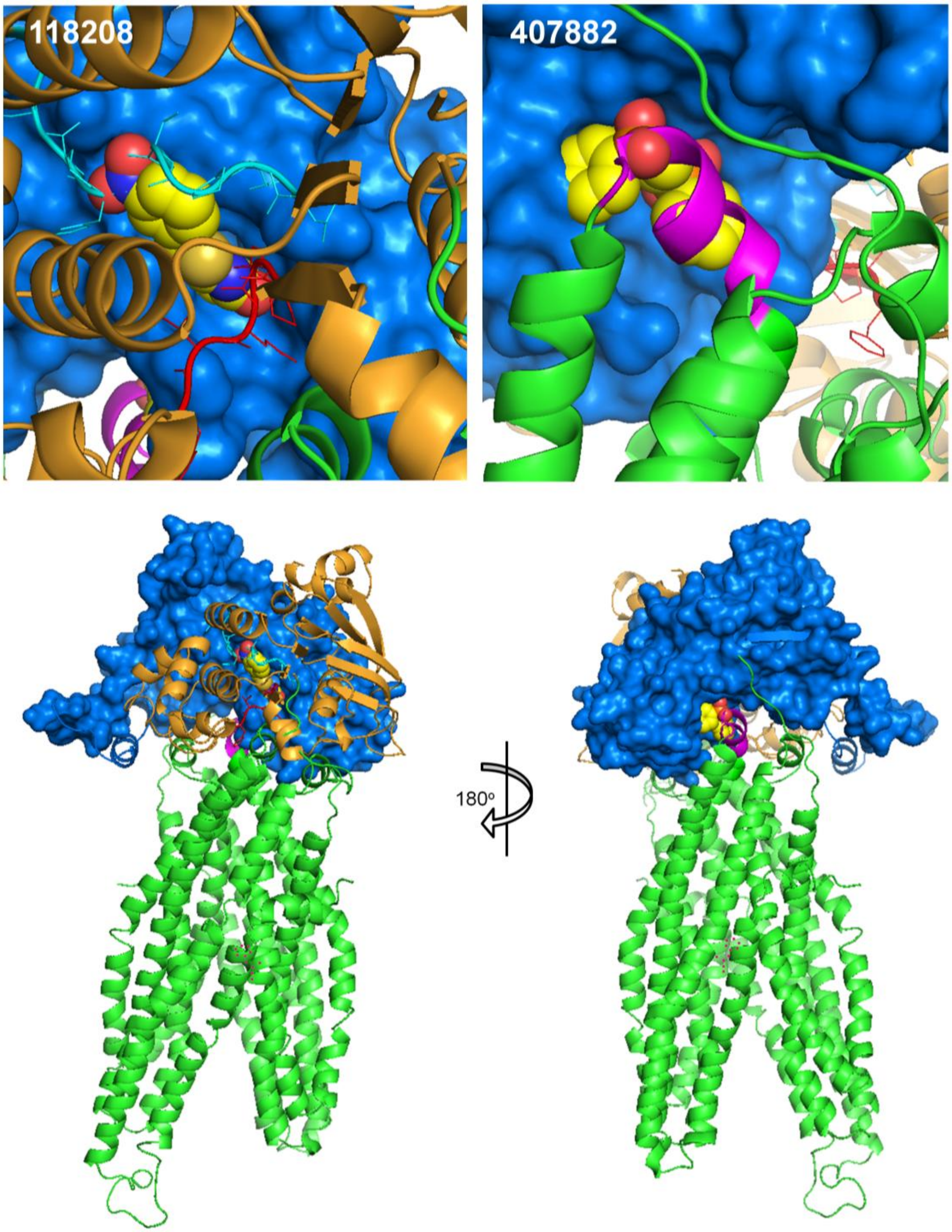


**Supporting Information Fig 7.** The full-length CFTR model coordinates have been adopted from Mornon at al. (Mornon et al, 2008). The TMD's and NBD2 have been represented by cartoon method colored green and orange respectively, whereas superimposed ΔF508-NBD1

“docking frame” has been shown as blue solvent accessible area. Two ligands 1108208 and 407882 have also been indicated.

The ICL4 of the TMD2, which in full-length CFTR model occupies pocket 2 is colored magenta, whereas loops P1290-F1294 (connecting two beta-strands F1286-P1290 and S1297-T1299), and E1371-D1377 (connecting β-strand I1366-D1370 with α-helix P1378-K1389) of NBD2 occupy pocket 1 region have been colored red an cyan respectively.

# Supporting Information Tables

## Supporting Information Table 1.

| Pocket 1 | | | | Pocket 2 | | | |
|---|---|---|---|---|---|---|---|
| NSC number | Chemical structure | Molecular weight | Solvent | NSC number | Chemical structure | Molecular weight | Solvent |
| **37173** | 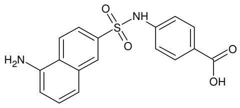 (1) | 531.10 | DMSO | **73100** | 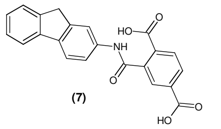 (7) | 373.36 | DMSO |
| **11668** | 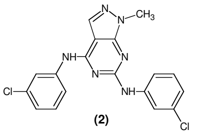 (2) | 385.25 | DMSO | **299589** | 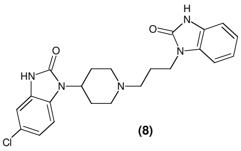 (8) | 425.92 | DMSO |
| **130813** | 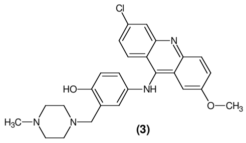 (3) | 462.98 | DMSO | **11237** | 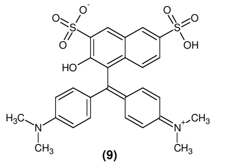 (9) | 554.63 | DMSO |
| **9608** | 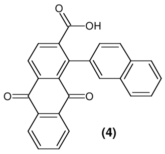 (4) | 378.38 | DMSO | **123526** | 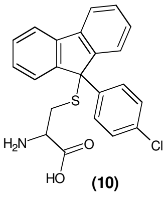 (10) | 395.90 | DMSO |
| **140873** | 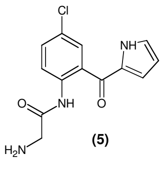 (5) | 277.71 | DMSO | **105687** | 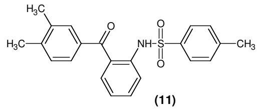 (11) | 379.47 | DMSO |
| **118208** | 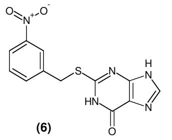 (6) | 303.29 | DMSO | **407882** | 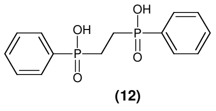 (12) | 310.23 | $H_2O$ |

DMSO – dimethyl sulfoxide

**Supporting Information Table 2.** Virtual Screening results from each scoring function for selected ligands

| NSC | TOTAL_E | VDW_E | ELE_E | G_SC | PMF_SC | D_SC | CHEMS | HPSc | HMSc | HSSc | Ave |
|---|---|---|---|---|---|---|---|---|---|---|---|
| | | | | | **POCKET 1** | | | | | | |
| **37173** | -46.298 | -16.071 | -30.228 | -118.95 | -1.99 | -91.58 | -28.84 | 5.50 | 5.47 | 5.76 | 5.58 |
| **11668** | -93.174 | -16.408 | -76.766 | -168.46 | -45.85 | -137.12 | -24.81 | 5.25 | 5.59 | 5.44 | 5.42 |
| **130813** | -76.193 | -7.318 | -68.875 | -105.29 | -12.93 | -101.54 | -27.08 | 5.23 | 5.54 | 5.37 | 5.38 |
| **9608** | -36.166 | -23.463 | -12.704 | -179.06 | -19.87 | -107.82 | -26.96 | 5.91 | 6.36 | 6.74 | 6.34 |
| **140873** | -37.654 | -13.256 | -24.398 | -76.63 | -9.48 | -82.51 | -28.76 | 5.88 | 6.32 | 6.17 | 6.12 |
| **118208** | -51.297 | -22.137 | -29.160 | -121.12 | -10.05 | -114.80 | -29.16 | 5.83 | 6.28 | 6.86 | 6.32 |
| | | | | | **POCKET 2** | | | | | | |
| **73100** | -61.571 | -20.245 | -41.326 | -65.81 | -1.93 | -108.05 | -35.53 | 6.02 | 6.04 | 6.40 | 6.15 |
| **299589** | -31.524 | -21.496 | -10.028 | -75.79 | -63.00 | -141.00 | -34.30 | 6.03 | 6.79 | 5.79 | 6.21 |
| **11237** | -32.048 | -24.454 | -7.594 | -102.44 | -39.26 | -81.22 | -32.42 | 5.74 | 6.39 | 5.77 | 5.97 |
| **123526** | -48.964 | -24.614 | -24.350 | -185.71 | -20.25 | -81.05 | -28.50 | 5.50 | 6.04 | 5.68 | 5.74 |
| **105687** | -70.663 | -19.515 | -51.148 | -178.76 | -39.14 | -95.70 | -26.59 | 5.47 | 6.58 | 5.36 | 5.80 |
| **407882** | -64.827 | -9.283 | -55.544 | -107.07 | -20.90 | -86.53 | -26.49 | 5.06 | 5.07 | 5.30 | 5.14 |

**Supporting Information Table 3.** Peptides with diminished HDex rates in the presence of correcting compounds.

| Compound | Peptides in pocket 1 | Peptides in pocket 2 | Peptides outside pockets |
|---|---|---|---|
| 407882 | 654-SAERRNSIL-662 | 505-NIIGVS-511<br>510-VSYDE-514 | 579-DVLTE-583<br>579-DVLTEKE-585 |
| 118208 | 526-LEEDISKFAEKDNIVLGEGGITLSGGQRARISL-558<br>527-EEDISKFAEKDNIVLGEGGITLSGGQRARISL-558<br>538-NIVLGEGGITLSGGQRARISL-558<br>542-GEGGITLSGGQRARISL-558 | | 579-DVLTE-583[A]<br>468-LMMIM-472 |

[A] this peptide increased but not sufficiently to be comprised ± 1 σ area.

**Supporting Information Table 4.** Peptides of WT-NBD1 with diminished HDex rates in the presence of correcting compounds.

| Compound | Peptides in Pocket 1 | Peptides in Pocket 2 | Peptides outside pockets |
|---|---|---|---|
| 407882 | | 505-NIIFG-509<br>505-NIIFGVS-511<br>559-ARAVYKDAD-567<br>559-ARAVYKDADLYL-570 | 579-DVLTE-583 |
| 118208 | | | 468-LMMIM-472<br>447-KIERGQL-453 |

**Supporting Information Table 5.** DynamX criteria for filtering the list of ΔF508-NBD1 peptides from PLGS program

| | |
|---|---|
| Minimum Intensity | 2000 |
| Minimum sequence length | 0 |
| Minimum products | 5 |
| Minimum products per amino acid | 0 |
| Minimum Consequitive Products | 2 |
| Minimum sum intensity for products | 0 |
| Minimum score | 0 |
| Maximum MH+ error (in ppm) | 10 |
| File threshold | 0 |
| Retention time RSD | -1% |
| Intensity RSD | -1% |

**Supporting Information Table 6.** Parameters for processing MS spectras by DynamX program

| | |
|---|---|
| Chromatographic Peak Width (min): | automatic |
| MS ToF Resolution | automatic |
| Lock Mass for charge 1 | 556,277 |
| Lock Mass for charge 2 | |
| Lock Mass Window | 0,25 |
| Low Energy Threshold | 40 |

## Supporting Information References

Antigny F, Norez C, Becq F, Vandebrouck C (2008) Calcium homeostasis is abnormal in cystic fibrosis airway epithelial cells but is normalized after rescue of F508del-CFTR. Cell calcium 43**:** 175-183

Baudouin-Legros M, Colas J, Moriceau S, Kelly M, Planelles G, Edelman A, Ollero M (2012) Long-term CFTR inhibition modulates 15d-prostaglandin J2 in human pulmonary cells. The international journal of biochemistry & cell biology 44**:** 1009-1018

Becq F, Mettey Y, Gray MA, Galietta LJV, Dormer RL, Merten M, Metaye T, Chappe V, Marvingt-Mounir C, Zegarra-Moran O et al (1999) Development of substituted benzo[c]quinolizinium compounds as novel activators of the cystic fibrosis chloride channel. J Biol Chem 274**:** 27415-27425

Bensalem N, Ventura AP, Vallee B, Lipecka J, Tondelier D, Davezac N, Dos Santos A, Perretti M, Fajac A, Sermet-Gaudelus I et al (2005) Down-regulation of the anti-inflammatory protein annexin A1 in cystic fibrosis knock-out mice and patients. Mol Cell Proteomics 4**:** 1591-1601

Crespin S, Bacchetta M, Huang S, Dudez T, Wiszniewski L, Chanson M (2011) Approaches to study differentiation and repair of human airway epithelial cells. Methods Mol Biol 742**:** 173-185

Eldridge MD, Murray CW, Auton TR, Paolini GV, Mee RP (1997) Empirical scoring functions: I. The development of a fast empirical scoring function to estimate the binding affinity of ligands in receptor complexes. J Comput Aided Mol Des 11**:** 425-445

Halgren TA (1999) MMFF VI. MMFF94s option for energy minimization studies. J Comput Chem 20**:** 720-729

Herrmann H, Wedig T, Porter RM, Lane EB, Aebi U (2002) Characterization of early assembly intermediates of recombinant human keratins. Journal of structural biology 137**:** 82-96

Hess B, Kutzner C, van der Spoel D, Lindahl E (2008) GROMACS 4: Algorithms for Highly Efficient, Load-Balanced, and Scalable Molecular Simulation. J Chem Theory Comput 4**:** 435-447

Hinzpeter A, Lipecka J, Brouillard F, Baudoin-Legros M, Dadlez M, Edelman A, Fritsch J (2006) Association between Hsp90 and the ClC-2 chloride channel upregulates channel function. Am J Physiol Cell Physiol 290**:** C45-56

Humphrey W, Dalke A, Schulten K (1996) VMD: visual molecular dynamics. J Mol Graph 14**:** 33-38, 27-38

Jones G, Willett P, Glen RC, Leach AR, Taylor R (1997) Development and validation of a genetic algorithm for flexible docking. J Mol Biol 267**:** 727-748

Jungas T, Motta I, Duffieux F, Fanen P, Stoven V, Ojcius DM (2002) Glutathione levels and BAX activation during apoptosis due to oxidative stress in cells expressing wild-type and mutant cystic fibrosis transmembrane conductance regulator. J Biol Chem 277**:** 27912-27918

Kuntz ID, Blaney JM, Oatley SJ, Langridge R, Ferrin TE (1982) A geometric approach to macromolecule-ligand interactions. J Mol Biol 161**:** 269-288

Kupniewska-Kozak A, Gospodarska E, Dadlez M (2010) Intertwined structured and unstructured regions of exRAGE identified by monitoring hydrogen-deuterium exchange. J Mol Biol 403**:** 52-65

Marivingt-Mounir C, Norez C, Derand R, Bulteau-Pignoux L, Nguyen-Huy D, Viossat B, Morgant G, Becq F, Vierfond JM, Mettey Y (2004) Synthesis, SAR, crystal structure, and biological evaluation of benzoquinoliziniums as activators of wild-type and mutant cystic fibrosis transmembrane conductance regulator channels. J Med Chem 47**:** 962-972

Meng EC, Shoichet BK, Kuntz ID (1992) Automated Docking with Grid-Based Energy Evaluation. J Comput Chem 13**:** 505-524

Mornon JP, Lehn P, Callebaut I (2008) Atomic model of human cystic fibrosis transmembrane conductance regulator: membrane-spanning domains and coupling interfaces. Cell Mol Life Sci 65**:** 2594-2612

Moustakas DT, Lang PT, Pegg S, Pettersen E, Kuntz ID, Brooijmans N, Rizzo RC (2006) Development and validation of a modular, extensible docking program: DOCK 5. J Comput Aided Mol Des 20**:** 601-619

Muegge I, Martin YC (1999) A general and fast scoring function for protein-ligand interactions: a simplified potential approach. J Med Chem 42**:** 791-804

Rabeh WM, Bossard F, Xu H, Okiyoneda T, Bagdany M, Mulvihill CM, Du K, di Bernardo S, Liu Y, Konermann L et al (2012) Correction of both NBD1 energetics and domain interface is required to restore DeltaF508 CFTR folding and function. Cell 148**:** 150-163

Richards FM (1977) Areas, volumes, packing and protein structure. Annu Rev Biophys Bioeng 6**:** 151-176

Sermet-Gaudelus I, Girodon E, Roussel D, Deneuville E, Bui S, Huet F, Guillot M, Aboutaam R, Renouil M, Munck A et al (2010) Measurement of nasal potential difference in young children with an equivocal sweat test following newborn screening for cystic fibrosis. In Thorax pp 539-544.

Tanguy G, Drevillon L, Arous N, Hasnain A, Hinzpeter A, Fritsch J, Goossens M, Fanen P (2008) CSN5 binds to misfolded CFTR and promotes its degradation. Biochim Biophys Acta 1783**:** 1189-1199

Wang R, Lai L, Wang S (2002) Further development and validation of empirical scoring functions for structure-based binding affinity prediction. J Comput Aided Mol Des 16**:** 11-26

Wieczorek G, Zielenkiewicz P (2008) DeltaF508 mutation increases conformational flexibility of CFTR protein. J Cyst Fibros 7**:** 295-300
